# Assessment of greenness for the determination of voriconazole in reported analytical methods

**DOI:** 10.1039/d1ra08858k

**Published:** 2022-02-28

**Authors:** Hemanth Kumar Chanduluru, Abimanyu Sugumaran

**Affiliations:** SRM College of Pharmacy, SRM Institute of Science and Technology Kattankulathur 603203 India abipharmastar@gmail.com abimanys@srmist.edu.in +91 7904062599

## Abstract

Analytical research with adverse environmental impact has caused a severe rise in concern about the ecological consequences of its strategies, most notably the use and emission of harmful solvents/reagents into the atmosphere. Nowadays, industries are searching for the best reproducible methods. Voriconazole is a second-generation azole derivative used effectively in the treatment of *Candida* and *Aspergillus* species infections and oropharyngeal candidiasis in AIDS patients. Recently it has become the drug of choice in treating mucormycosis in several countries, which raises the need for production in large quantities. The present review deals with various recent important analytical techniques used to estimate voriconazole and its combination in pharmaceutical formulations and biological fluids. The methods show their own unique way of analyzing voriconazole in different matrices with excellent linearity, detection, and quantification limits. Additionally, this article deals with methods and solvents analyzed for their impact on the environment. This is followed by estimating the degree of greenness of the methods using various available assessment tools like analytical eco-scale, national environmental method index, green analytical procedure index, and AGREE metrics to confirm the environmental impact. The scores obtained with the evaluation tools depict the quantum of greenness for the reported methods and provide an ideal approach adopted for VOR estimation. Very few methods are eco-friendly, which shows that there is a need for the budding analyst to develop methods based on green analytical principles to protect the environment.

## Abstract 2

This report assessed the greenness profiles of various determination methods for voriconazole in bulk and pharmaceutical dosage forms using various assessment tools. The types of solvents utilized in the analysis were examined as well.

## Graphical abstract

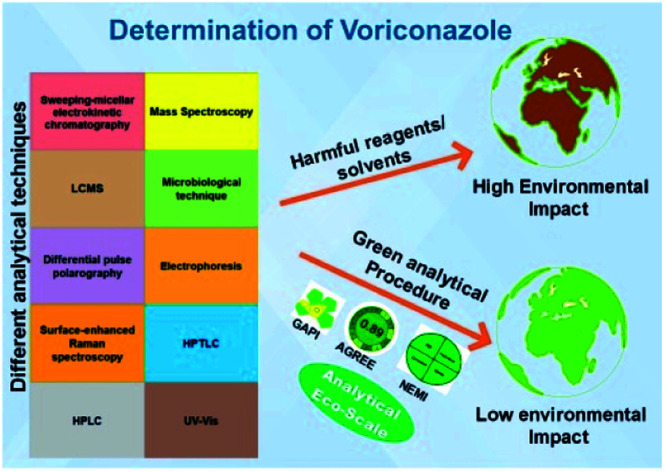

## 1. Introduction

Developing analytical methods to determine medicines in bulk or pharmaceutical dose form is never an easy endeavour due to chemical complexity and variety. Many attempts have been made by analytical scientists from a variety of sectors to produce an individual robust analytical technique that is also compliant with regulatory requirements. Regulators are increasingly seeking eco-friendly techniques to reduce or eliminate the formation of harmful effluents due to pollution created by analytes, solvents, and chemicals used in analytical departments. It is the purpose of this study to discuss different modern and essential analytical methods that have been developed to estimate voriconazole and its combination in pharmaceutical formulations and biological fluids, as well as the degree of greenness of the same.

### 1.1. Voriconazole

**Fig. 1:**
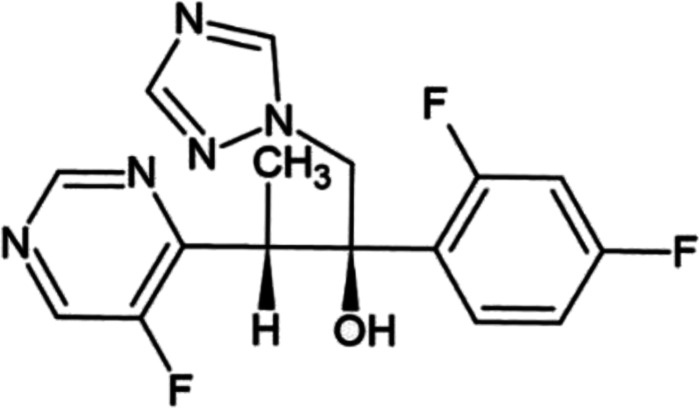
Structure of voriconazole.

Voriconazole (VOR) is chemically (2*R*,3*S*)-2-(2,4-difluorophenyl)-3-(5-fluoro-4-pyrimidinyl)-1-(1*H*-1,2,4-triazol-1-yl)-2-butanol (Fig. 1). It works by inhibiting the fungal 14-alpha-lanosterol demethylation cytochrome P450-dependent 14-sterol demethylase, a vital enzyme in ergosterol biogenesis required to form fungal cell walls.^1–4^

VOR has become an essential drug for treating mucormycosis or black fungus in the initial stages of their existence as well as SARS-CoV-2. VOR is also used for people diagnosed with *Candida* and *Aspergillus* species infection, which have death rates of more than 60%. In addition, they are more likely to develop candidemia, leading to resistance to triazole antifungal drugs if not appropriately treated.^5^ While there are several treatment alternatives, currently available antifungal medicines do not meet the needs of many patients, especially those who take their medication by oral administration or through intravenous injection. Voriconazole (VOR) is the most critical triazole antifungal drug to enter the arsenal of antifungal agents. It has a structure similar to that of fluconazole and an activity spectrum comparable to that of itraconazole. In May 2002, the FDA approved VOR to treat *Fusarium* species refractory *Scedosporium apiospermum* and invasive aspergillosis infections. VOR has also been shown to be a promising drug for empiric treatment of febrile neutropenia in studies.^6^ The Jing Wang *et al.* study revealed that VOR is effectively used as the best prophylaxis option for patients undergoing hematopoietic stem cell transplantation.^7^ Voriconazole has high bioavailability (96%) and has been shown to penetrate various eye areas, with adequate concentrations obtained to cover a wide variety of keratitis-causing fungi. Voriconazole eye drops, produced ad hoc and then used off-label, have been recommended successfully to treat keratitis. Voriconazole showed adequate penetration *via* the cornea into the aqueous humour after topical treatment without affecting intraocular tolerability.^8^

**Fig. 2:**
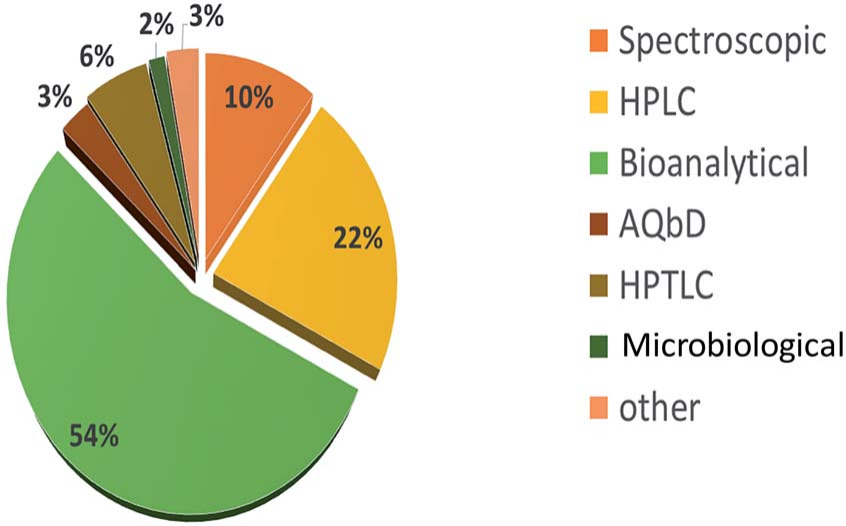
Methods available for the estimation of VOR.

**Fig. 3:**
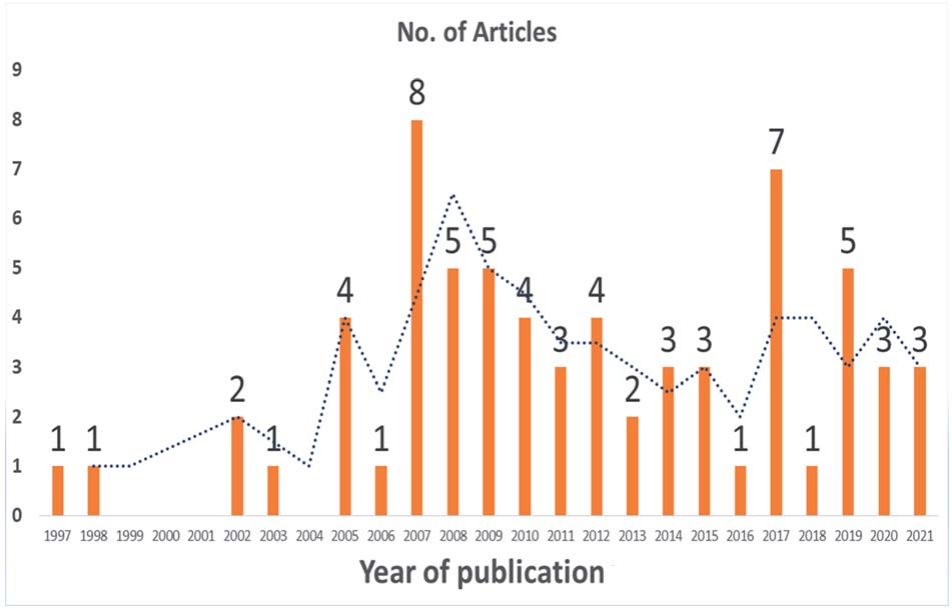
Analytical methods reported for VOR from 1997 to September 2021 (source: Google, PubMed, Taylor and Francis, Elsevier, Science Direct, and Scopus).

VOR is available in different dosage forms for the treatment of many fungal infections. Analytical techniques are used throughout the drug development process, from pre-clinical to post-clinical testing, to understand the drug's physical and chemical stability, impact on dosage form selection and design, and quantification of impurities. Various technological enhancements in separation science, modern sophisticated spectroscopic and liquid chromatographic techniques, and the use of bioanalytic tools for molecular recognition and testing have been of great advantage to pharmaceutical analysis in recent years. The whole dosage form development process requires effective, precise analytical procedures to support every step. Pharmaceutical companies are obliged to use the most accurate, prudent, and dependable quality control methods to quantify VOR. The reported best analytical methods for estimating VOR present in different pharmaceutical dosage forms are highlighted to illustrate the importance of VOR analyses (Fig. 2 and 3).

Analytical methods such as chromatographic techniques utilize toxic solvents and have a deleterious effect on the environment.

### 1.2. Effect of analytical methods on the environment

Liquid chromatography and its related methods are widely used and accepted for the estimation of pharmaceutical substances. The mobile phase commonly employs components such as water, buffers, additives to adjust pH, and organic modifiers like methanol (MeOH) and acetonitrile (ACN). These organic modifiers are mostly preferred for the LC method because of their ease of practical applicability as well as miscibility in water, low UV wavelength cut-off range (205 nm for MeOH and 190 nm for ACN), high purity, and low or no reactivity with most pharmaceutical substances.^9,10^ Despite the remarkable advantages of liquid chromatography, most reported solvents have a highly adverse environmental impact.^11,12^ ACN causes adverse health effects since it is a systemic irritant, is flammable, toxic, and volatile, and is categorized as an occupational hazard for analysts due to risk of inhalation and skin/eye contact. Methanol's vapor pressure is low; thus, it can easily volatilize into the surrounding air. Methanol degrades through interaction with airborne hydroxyl radicals after volatilization, and its half-life is about 18 days.^13^ Organic solvents like MeOH and ACN that are frequently used in chromatographic analysis affect animals, birds, and fish, cause death, and affect biota fertility. These two solvents are also included in the EPA's (Environmental Protection Act) TRI (Toxicity Reactivity Ignitability) list as hazardous solvents.

RP-HPLC consists of a stationary phase (column) and mobile phase (solvents) with a flow rate of 0.8–1.5 mL min^−1^. On average, usage of HPLC on a single day with a 50 : 50 ratio of organic phase and buffer with 1 mL min^−1^ flow rate generates 1.5 L/24 h (750 mL of organic waste and 750 mL of aqueous buffer waste containing toxic chemicals in a single working day). Developing an eco-friendly method by controlling the waste generated without affecting the method quality and performance for analyzing compounds in HPLC is a mammoth task. However, this problem can be reduced by applying green analytical principles in drug analysis. This particular review aims to summarize and examine various VOR estimation methodologies currently available using different instrumental methods along with the estimation of the degree of the greenness of the same.

Anastas^14^ portrayed 12 green analytical chemistry principles from the general green chemistry principles to help analysts in developing an environmentally fit method that can be used in the long term without affecting the environment.

### 1.3. Green analytical chemistry principles^15–20^

Green analytical chemistry principles are derived from the modification of green chemistry principles. Each principle has its unique role in the development of an analytical method. However, it is impractical to apply all the principles, but the number of principles incorporated in the method development makes the output most eco-friendly. (1) The generation of hazardous waste in sampling can be reduced by eliminating large volume dilutions. (2) The direct sampling technique cannot be implemented to analyze all samples in liquid chromatography due to its limitation. Still, this principle can be satisfied by selecting other techniques like IR and FTIR to make the analysis eco-friendlier. (3) Green or biodegradable chemicals or solvents must be used instead of toxic chemicals in the environment, *e.g.*, methanol; acetonitrile needs to be replaced by ethanol and propylene carbonate. (4) Renewable solvents like 1,1-diethoxyethane, isosorbide dimethyl ether, eucalyptol, rose oxide, γ-terpinene, and α-pinene^21^ shall be replaced by the other toxic solvents for the analysis. (5) The waste generation in liquid chromatography (LC) techniques has a significant environmental impact and is inevitable. Instead, the generated waste can be recycled using different distillation processes. (6) Upgrading existing techniques by miniaturization, wherein a lab-on-a-chip miniature device is developed, is a sophisticated technique for analyzing compounds in a simple step. (7) The novel combination of molecular biology with microelectronics has resulted in the electronic detection of biomolecules through field-effect transistors (FETs) and lab-on-a-chip biosensors.^22^ (8) Multiple analytical techniques have to be applied for the new method development. (9) Energy consumption for analyzing a sample should be as less as possible to make the method greener; for example, the application of UPLC rather than HPLC uses less energy. (10) Derivatization is an extra step for analyzing a drug that should always be avoided in most cases but it is an inevitable step; then, the green reagents need to be utilized for this step *e.g.*, nicotinic acid, hydrindantin dihydrate, ferrocene carboxaldehyde, and (+)-diacetyl-l-tartaric anhydride. (11) *In situ* measurements necessitate that equipment is placed directly at the site of analysis and in touch well with the subject of interest of the drug or sample. (12) Occupational hazards need to be nullified in consideration towards the analyst.

As it is mentioned early that application of all the principles in analytical methods is practically very difficult so some strategies have to be applied for developing the methods that should be environmentally safe.

### 1.4. Strategies for greening an analytical process – solvent reduction^23–28^

Reduction of solvent consumption leads to the reduction of waste. There are numerous ways to reduce it. (i) Using RP-HPLC methods instead of the normal phase will allow for polar solvents that are non-hazardous. (ii) Short column usage will make the elution faster and reduce waste. (iii) Microflow and capillary HPLC columns will decrease the flow rates and reduce solvent consumption. (iv) Higher column temperature will decrease water viscosity and also enhances polar characteristic that reduces the use of organic modifiers. (v) Newer columns like fused core particle columns will have smaller particle sizes that makes the separation faster and better. (vi) Finally, miniaturization from HPLC to UPLC with short columns and high pressure makes the analysis faster and decreases the waste generated.

#### 1.4.1. Solvent replacement^29–33^

Flammable and toxic solvents can be replaced by bio solvents, as follows: (i) toxic solvents such as ACN, MeOH, and ethyl acetate can be replaced by eco-friendly solvents such as propylene carbonate, ethanol, and ethyl lactate, respectively. These eco solvents have similar properties to the other solvents and are a perfect replacement. (ii) Superheated water at 80–250 °C can be an alternative in specific cases as water is inexpensive, non-flammable, eco-friendly and has a low UV cut-off wavelength. However, this has drawbacks, such as the fact that hydrophobic samples cannot be analyzed by simple water and thermolabile drugs cannot be determined at these high temperatures. (iii) Supercritical fluid can also be a better substitute as it provides higher separation and faster elution.

#### 1.4.2. Sample preparation^34–38^

Choosing a direct analytical method is not possible for analyzing all samples in LC. Instead, sample preparation can be done by (i) using eco-friendly solvents for preparation of samples by avoiding transportation and (ii) unavoidable extraction processes using toxic chemicals can be replaced by micro-wave assisted, ultrasound-assisted, and pressurized liquid extraction.

#### 1.4.3. Analytical quality by design (AQbD)^39–43^

The QbD approach suggests looking into the quality of the analytical process during the development stage itself. It says that quality should be built into the process design rather than testing into results of the analytical process. When the QbD principles are applied in the method development of pharmaceutical substances, the strategy can be called analytical quality by design. The outcome of AQbD is well understood and fit for its intended purpose with sturdiness throughout the lifecycle.

## 2. Evaluation of greenness

A method should be carefully assessed before claiming the greenness of the technique. However, GAC's lack of dedicated evaluation tools has been considered the main problem in greenness estimation. Although different evaluation tools are available for green chemistry, all have their limitations in GAC. A few green assessment tools that can be applied in GAC are as follows.

### 2.1. National environmental methods index (NEMI)^44–48^

NEMI is considered as the oldest evaluation tool used to assess green chemistry and represent a quadratic pictogram. Each quadra represents one factor: persistent bioaccumulative and toxic (PBT), hazardous, corrosive, and waste. If a chemical is used in the method listed in the EPA (Environmental Protection Act) TRI (Toxicity Reactivity Ignitability) list, the quadra must be left blank. If not listed, it should be coded with green color. Solvents/chemicals, if listed in Resource Conservation and Recovery Act (RCRA), were used in the method, then the next quadrant should be left blank; if the chemicals used in the process are non-corrosive, then the next quadrant should be coded green; finally, if the waste produced by the method is more than 50 g mL^−1^ then the next quadrant has to be left blank.

### 2.2. Green analytical procedure index (GAPI)^49–54^

This evaluation system is also similar to NEMI, but it covers a few more aspects. The hazardous solvents need be to checked in the National Fire Protection Act (NFPA) instead of the EPA TRI list for GAPI. The pictogram contains three colors: green, yellow, and red instead of green or none. Colour should be coded based on the solvents used, like highly toxic coded as red, moderately toxic as yellow, and less or non-toxic as green. Different parameters that need to be checked for the GAPI are sample preparation, preservation, transport, storage, type of method, the scale of extraction, solvents used, additional treatments, and amount of reagents used. Although it covers most aspects required to evaluate GAC, the output based on color representation makes this a qualitative rather than quantitative tool.

Płotka^55^ developed a new tool in 2021 named complementary GAPI or complex GAPI, which is an advancement of GAPI. This complex GAPI is a combination of classical GAPI and E-Factor, where E-Factor mainly focused on the synthesis of the chemicals along with the product yield, purity, waste, *etc.* Because the study design is solely concerned with the development of analytical methods and their greenness estimate, and there is no published literature on synthesis data of analyte and solvent, the implementation of the complex GAPI is minimal.

### 2.3. Analytical eco-scale (AES)^56,57^

Although eco-scale was first introduced to evaluate GC, it shows promising results when applied to GAC. When eco-scale is used for analytical methods, then it can be called AES. This tool has an output of numerical values as the eco-scale score equals ‘100’. The deduction of the eco score is based on the penalty points (PP) scored by the method.

PP for reagents used:

• Less than 10 g or mL = 1 PP.

• 10–100 g or mL = 2 PP.

• More than 100 g or mL = 3 PP.

PP based on chemicals used:

• Pictogram with danger representation = 2 PP.

• Pictogram with warning representation = 1 PP.

• No Pictogram representation = 0 PP.

PP based on energy used per sample.

• Less than or equal to 0.1 kW h = 0 PP.

• Less than or equal to 1.5 kW h = 1 PP.

• More than or equal to 1.5 kW h = 2 PP.

PP based on waste generated.

• No waste = 0 PP.

• Less than 10 g or mL = 1 PP.

• 1–10 g or mL = 3 PP.

• More than 10 g or mL = 5 PP.

The results of greenness from the eco scale.

• Eco score ≥ 75 = worthy green method.

• Eco score ≥ 50 = optimal green method.

• Eco score < 50 = not a green method.

### 2.4. Analytical GREEnness (AGREE)^58,59^

It is a software-based assessment tool covering all the 12 principles of GAC. It works based on graphical user interface (GUI) technology. It gives a result in numerical values with a high of 1 showing complete green, whereas the decrease in the number indicates a reduction in greenness. It contains 12 steps as the sampling procedure, sample size, *in situ* measurement, steps in the process, miniaturization, derivatization, waste, number of analytes, energy, type of reagents, toxic reagents, and operator safety need to be filled in software for obtaining the green results. The score of 1 indicates a complete green method, and the result near 1 indicates that the process was obeying the eco-friendly conditions and *vice versa*.

### 2.5. Analytical method greenness score (AMGS)^60–62^

AMGS is a spreadsheet calculator dedicated to the chromatographic method of analysis. It is an amalgamation of HPLC-environmental assessment tool cumulative energy demand (CED) for instrument and solvent selection, analytical mass volume intensity (AMVI) for assessing solvent wastage, and safety, health, and environmental assessment (SHE) for determining solvent safety through geometric mean. The present spreadsheet works by having the data of the method and executing it for the evaluation; the output value should be as low as possible to obtain the greenest of the results.

### 2.6. Carbon footprint

Carbon footprint is an important concept for calculating the emission of global warming gases into the atmosphere generated during any fossil fuel combustion. It is important to calculate the carbon footprint for an industry in a whole process to understand the overall emission of greenhouse gasses into the atmosphere. The present study deals with the analysis of voriconazole using different analytical instruments, as this equipment utilizes significantly fewer energies for the determination of single samples (HPLC consumes ≥1.5 kW h, UPLC and UV-VIS spectrophotometry absorbs <1.5 kW h, and LCMS utilizes >1.5 kW h). This energy consumption generates significantly less CO_2_, which produces negligible effects on the environment. This energy measurement was incorporated into a few greenness assessment metrics like GAPI, AES, and AGREE, which nullifies the complexity of the carbon footprint analysis regarding energy consumption.

The above assessment tools have been utilized to demonstrate the greenness in reported methods for the analysis of VOR to scrutinize and select the best approach in terms of eco-friendliness. Although each greenness assessment tool utilizes a different way of analyzing the greenness profile, the final results help to picturize and identify the more environmentally benign method with its environmental impact. Here, four assessment tools were used for assessing the greenness of the reported methods.

## 3. Methods for the quantification of VOR and its combinations

### 3.1. Official methods

VOR was added to the pharmacopeias such as the United States Pharmacopeia (USP), British Pharmacopeia (BP), and Indian Pharmacopeia (IP) officially^63–65^ in 2018 after 16 years of approval from the FDA. All these methods have used liquid chromatography as a technique for the analysis of VOR.

### 3.2. United States Pharmacopeia, British Pharmacopoeia, and Indian Pharmacopoeia

**Fig. 4:**
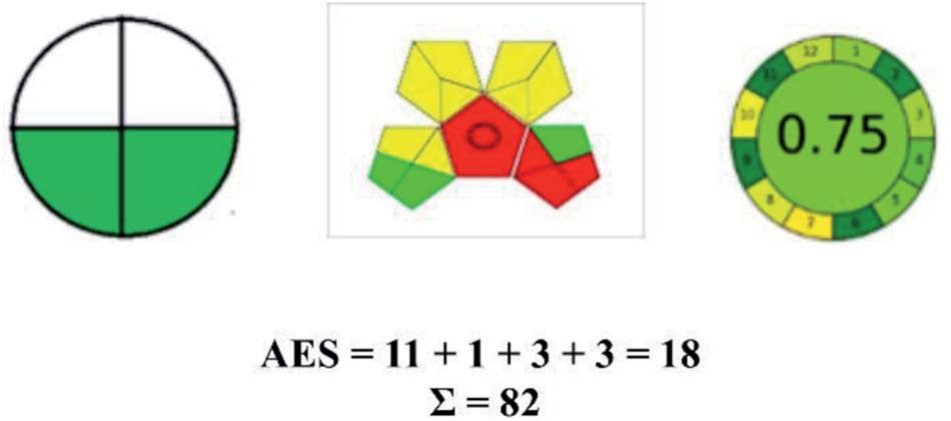
NEMI, GAPI, AES, and AGREE assessments for the official methods.

The major official pharmacopeias state that the method for the analysis of VOR was HPLC using an L1 packing column with end-capped octadecyl silyl silica gel (150 mm × 3.9 mm, 4 μm), with a mobile phase consisting of ACN : MeOH : ammonium formate (pH 4.0) in a ratio of 15 : 30 : 55 v/v/v at a flow rate of 1.0 mL min^−1^ determined in a wavelength of 256 nm with a retention time of 8 min for VOR. The acceptance criterion according to USP, BP, and IP for VOR was 97.5–102.0%. The greenness assessment for the official method was performed using the NEMI, GAPI, AES, and AGREE assessment tools for the pharmacopeia data and is depicted in Fig. 4.

### 3.3. Reported methods

Articles were collected for VOR in pharmaceutical dosage forms from Science Direct, PubMed, Scopus, Taylor and Frances, Google, and different web sources from 1997 to 2021 and were organized and executed for the present review.

#### 3.3.1. UV-VIS spectrophotometry

Spectroscopic methods like UV-VIS play a significant role in the quantification and qualification of most of the drugs. UV-VIS is essential in all quality control departments to make the analysis of drugs accurate and simple. It is also combined with modern analytical techniques like HPLC to give more accurate results. The determination of VOR by UV has used MeOH,^66,67^ water,^68^ HCL,^69^ and phosphate buffer^70^ in a wavelength range around 252–256. The visible range for the derivatization chemicals/solvents like tropaeoline ooo and azocarmine-G was used at a wavelength of 500 and 550 nm. The overall spectrophotometric methods reported for VOR analysis and the results of the assessment of greenness by applying four tools are depicted in Table 1.

**Table 1:** Spectroscopic techniques reported for determining VOR and their greenness assessment

S. no.	Matrix of VOR	Reagents or solvents	Detection (nm)	Linearity (μg mL^−1^)	NEMI	GAPI	AES	AGREE	Ref.
1	Bulk and tablet	MeOH	252	5–80	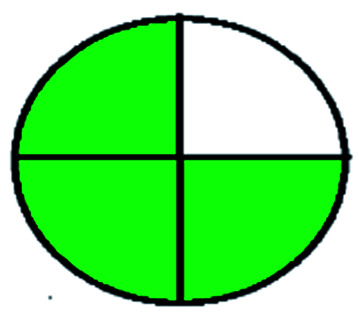	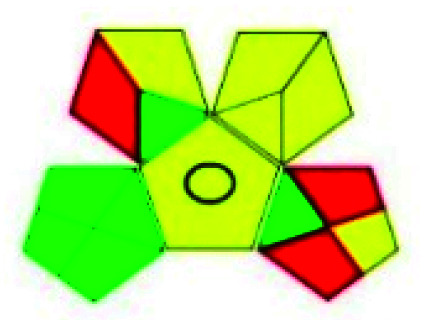	8 + 0 + 2 + 5 = 15	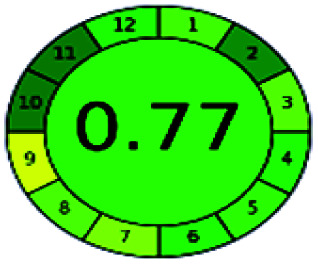	66
							*Σ* = 85		
2	Bulk and tablet	Milli Q pore water	255	5–35	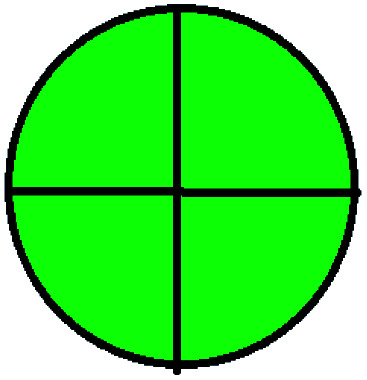	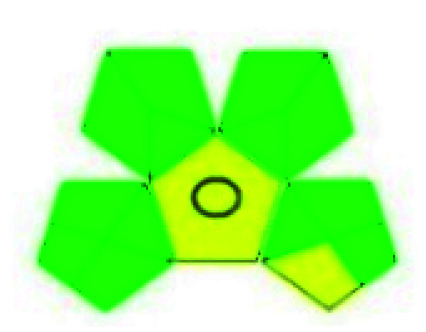	0 + 0 + 0 + 0 = 0	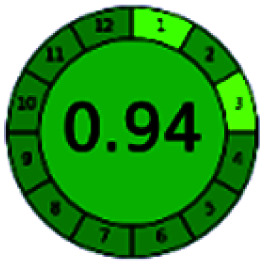	68
							*Σ* = 100		
3	Tablet	MeOH	256	5–30	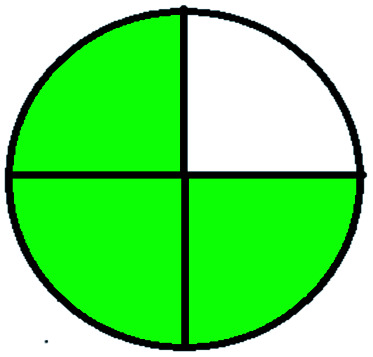	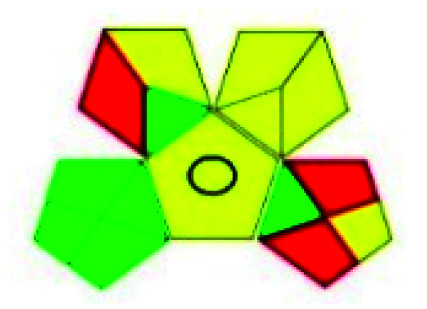	8 + 0 + 2 + 5 = 15	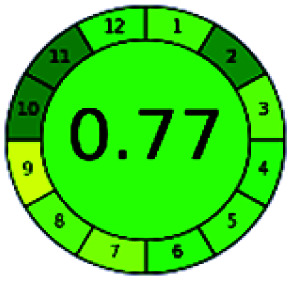	67
							*Σ* = 85		
4	Tablets	(1) 0.1 M HCl and tropaeoline ooo	(1) 500	(1) 5.0–2	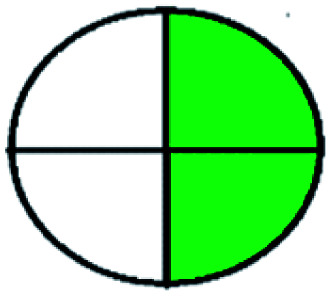	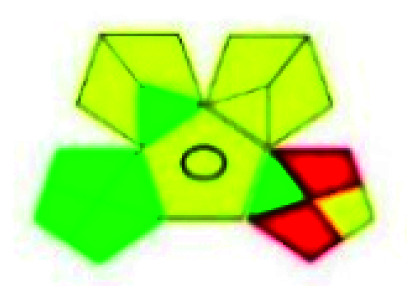	8 + 0 + 1 + 5 = 14	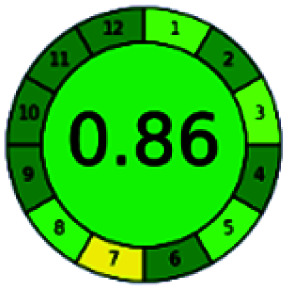	71
							*Σ* = 86		
		(2) pH 1.5 buffer and azo carmine-G	(2) 550	(2) 10–50	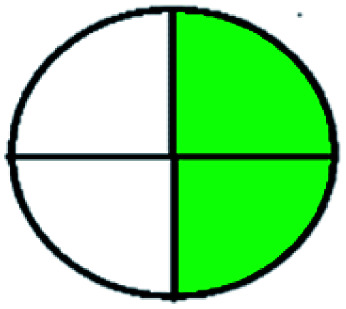	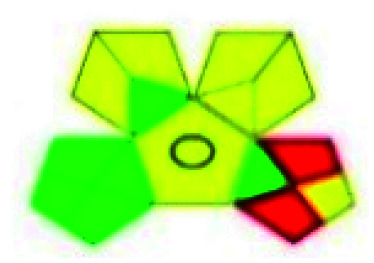	8 + 0 + 1 + 5 = 14	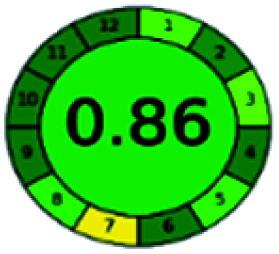	
							*Σ* = 86		
5	Bulk powder and pharmaceutical dosage form	0.1 N HCl	256	10–60	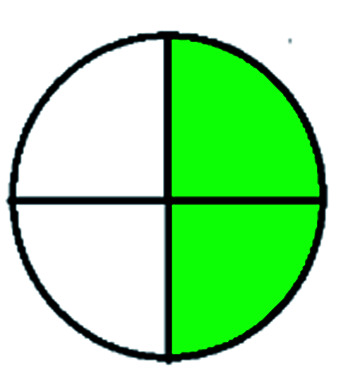	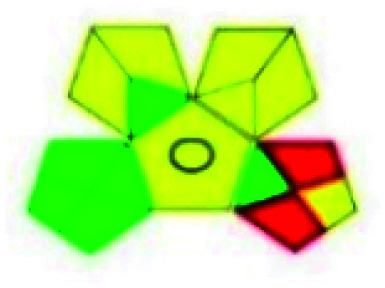	8 + 0 + 1 + 5 = 14	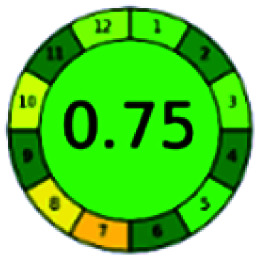	69
							*Σ* = 86		
6	Tablets	Phosphate buffers (pH 2.0, 4.0, 6.8, and 7.0)	200–400	5–60	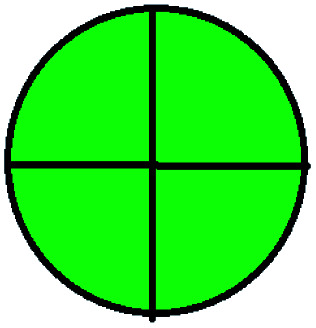	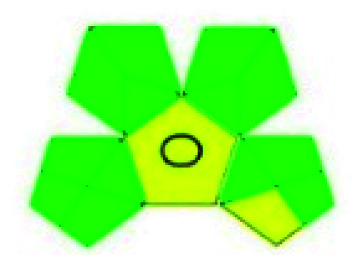	1 + 0 + 0 + 0 = 1	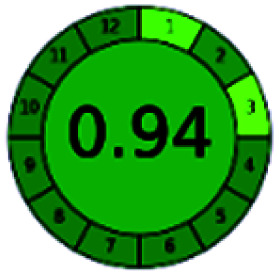	70
							*Σ* = 99		

#### 3.3.2. High-pressure liquid chromatography

HPLC is a critical and widely used analytical method for quantifying the majority of pharmaceutical dosage forms. Additionally, HPLC is a very trustworthy technique for measurement due to its precision, robustness, and sensitivity. Estimating VOR has utilized a set of different mobile phase combinations with a common organic phase like MeOH and ACN and various phosphate buffers. Initial development results showed the longest retention time of 21.06, but technology improvisation leads to the shortest elution of analytes at 3.02 min.^72^ Although the linearity selected in the reported HPLC methods did not broadly vary from the corresponding procedures, the chosen narrow range among the methods was 1 to 30.^73^ Detectors like UV and photo diode array (PDA) were considered as another important factor for more accurate analysis of drugs with the reported methods of RP-HPLC. Among the two detectors PDA was selected by most of the methods due to their advantages like fast and more sensitive detection at multiple wavelengths. Table 2 summarizes the RP-HPLC techniques reported for the VOR study in single and combination dosage forms along with the application of four green assessment tools.

**Table 2:** Greenness assessment of reported HPLC methods for determining VOR and its combinations^a^

S. no.	Drug substance	Stationary phase and mobile phase	Linearity (μg mL^−1^)	*R*_*t*_ (min)	NEMI	GAPI	AES	AGREE	Ref.
1	VOR	Merck LiChrospher 100 RP-8 (125 mm × 4.6 mm, 5 μm)	20–100	NA	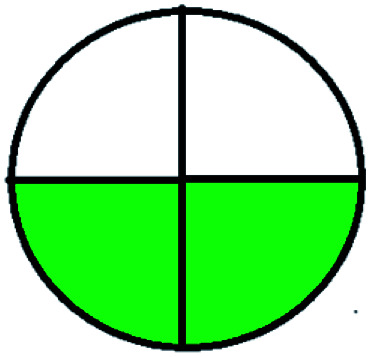	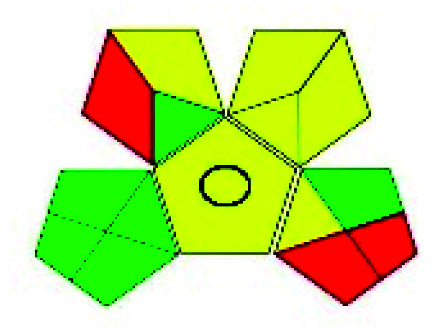	36 + 1 + 3 + 5 = 45	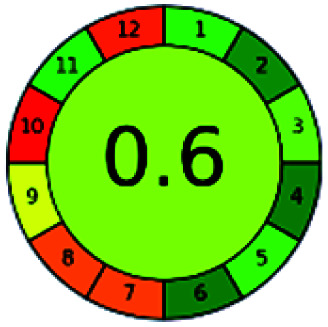	74
		0.6% triethylamine (pH 6.0) : MeOH (50 : 50 v/v)					Eco score = 55		
2	VOR	Chiral cel-OD (250 mm × 4.6 mm, 10 μm)	25–200	21.06	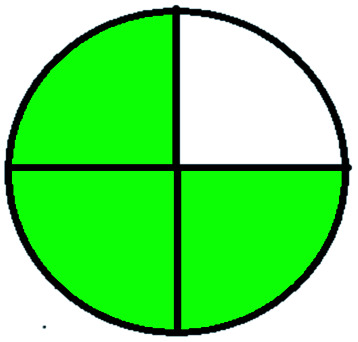	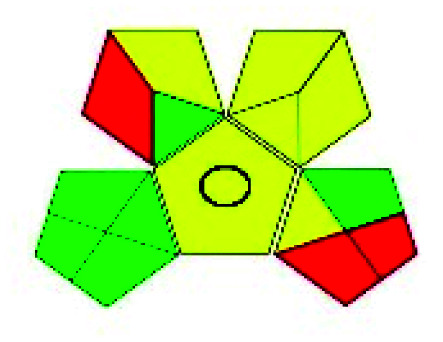	20 + 1 + 3 + 5 = 29	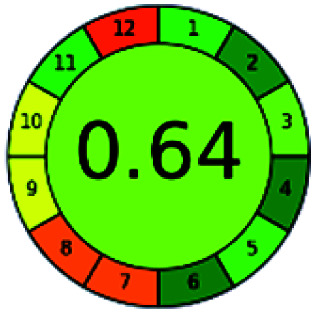	75
		*n*-Hexane : EtOH 9 : 1 (v/v)					ES = 71		
3	VOR	Diamonsil C18 column (250 mm × 4.6 mm, 5 μm)	1–100	14.002	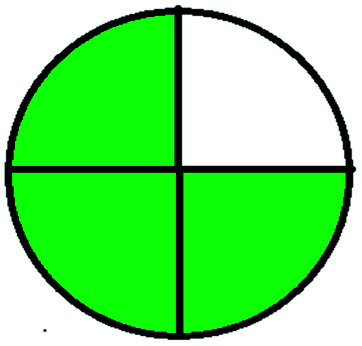	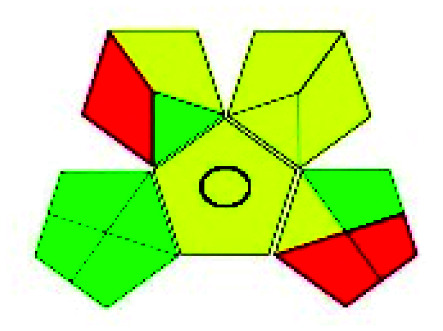	12 + 1 + 3 + 5 = 21	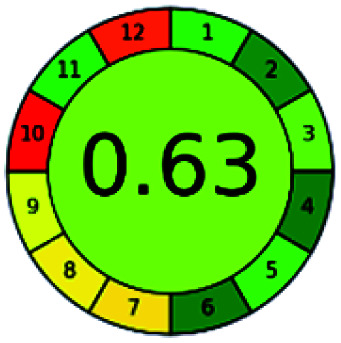	76
		ACN : water : CH_3_COOH (40 : 60 : 0.25 v/v/v)					ES = 79		
4	VOR	C-18 Hypersil BDS column (250 mm × 4.6 mm, 5 μm)	20–400	12.98	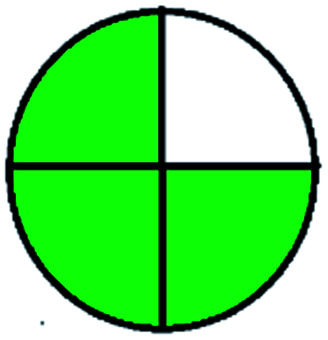	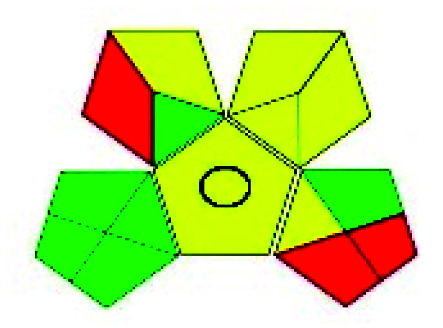	15 + 1 + 3 + 5 = 24	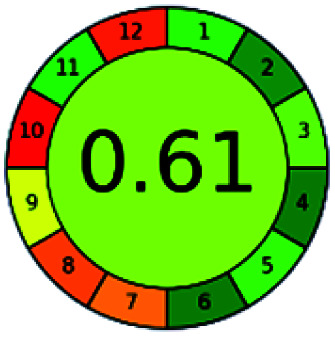	77
		Water : ACN : MeOH (50 : 25 : 25 v/v/v)					ES = 76		
5	VOR	Hypersil C18 (250 mm × 4.6 mm, 5 μm)	5–25	NA	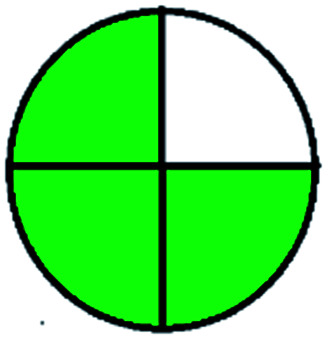	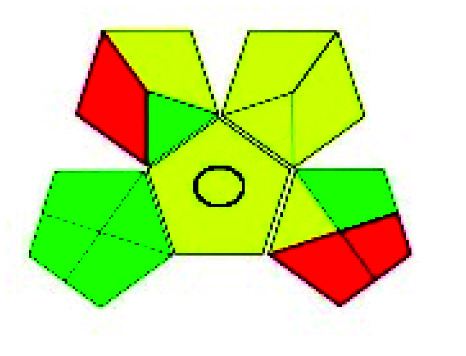	4 + 1 + 3 + 5 = 13	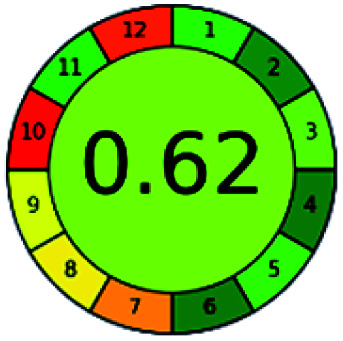	78
		ACN : water (40 : 60 v/v)					ES = 87		
6	VOR	Zorbax SB-C18 (250 mm × 4.6 mm, 5 μm)	10–100	6.7	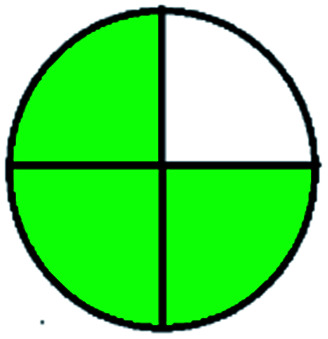	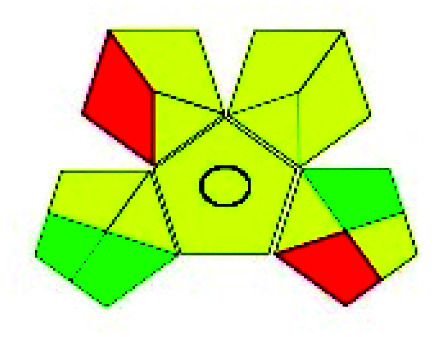	7 + 1 + 3 + 3 = 14	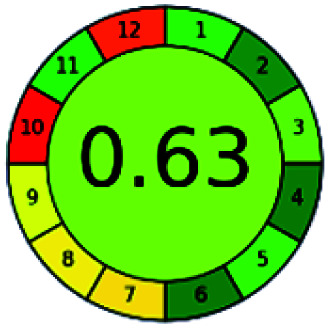	79
		Ammonium phosphate dibasic buffer (pH 6.0 with; 50 mM *ortho* phosphoric acid)–ACN (52 : 48 v/v)					ES = 86		
7	VOR	Hypersil, C18 (250 mm × 4.6 mm, 5 μm)	5–100	5.82	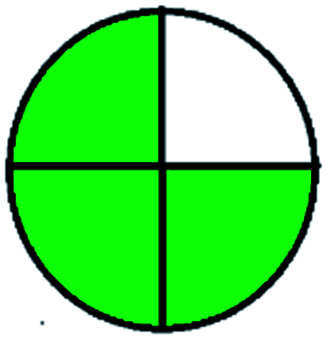	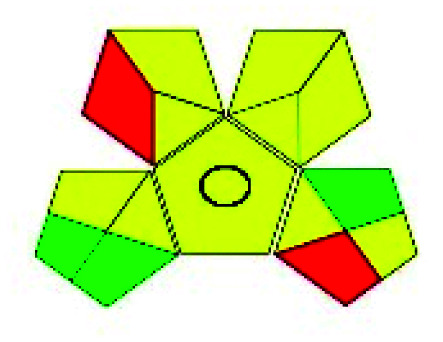	4 + 1 + 3 + 5 = 13	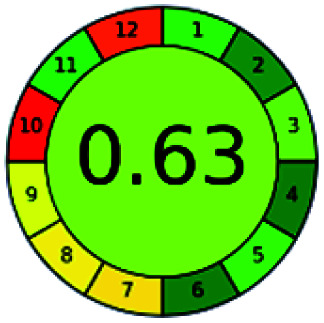	80
		Water : ACN (50 : 50 v/v)					ES = 87		
8	VOR	Bondapak C18 (10 μm, 250 mm × 4.6 mm)	6.0–60	7.3	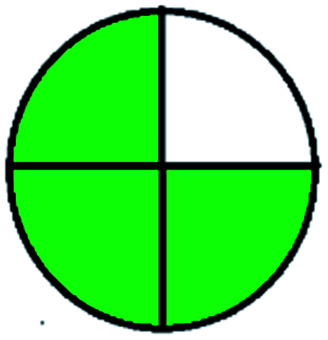	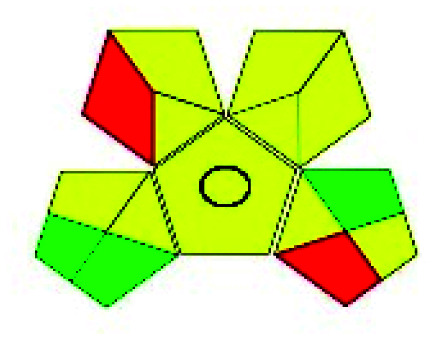	4 + 1 + 3 + 3 = 11	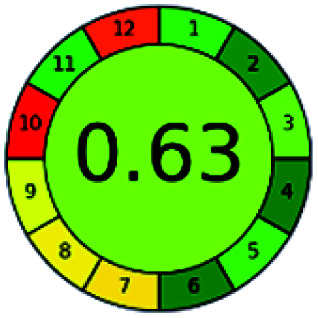	81
		ACN : 0.05 M disodium hydrogen phosphate buffer (pH 5.5) (1 : 1 v/v)					ES = 89		
9	VOR	Intersil ODS C18 (150 × 4.6 mm, 5 μm)	7.94–11.91 ppm	6.413	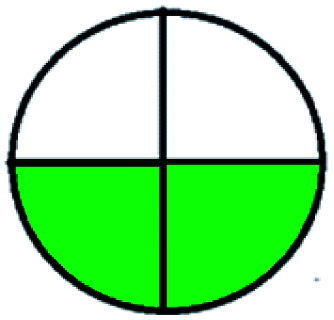	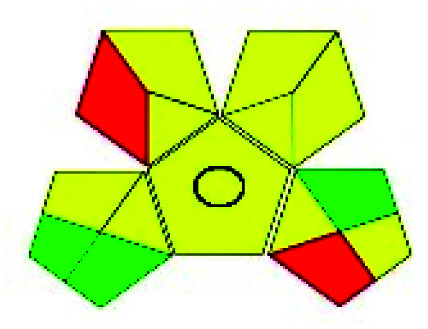	10 + 1 + 3 + 5 = 19	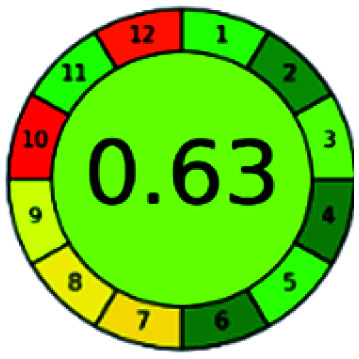	82
		Phosphate buffer, ACN and MeOH (65 : 30 : 5 v/v/v)					ES = 81		
10	VOR	C18 G column (250 mm × 4.6 mm, 5 μm)	10–50	5.36	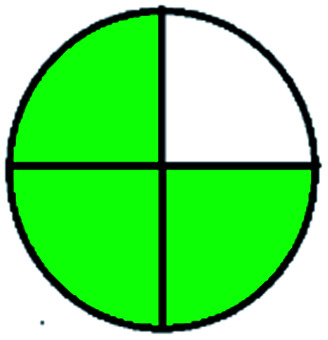	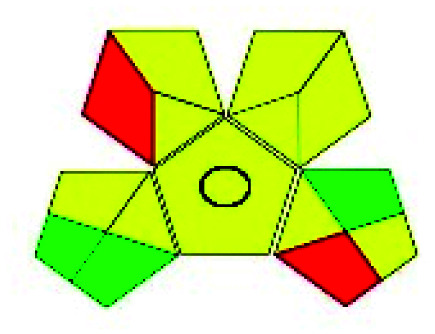	4 + 1 + 3 + 5 = 13	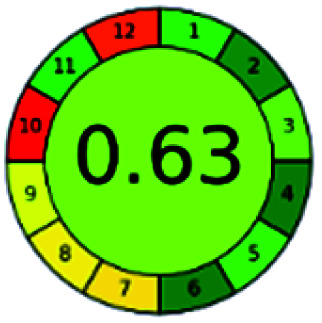	83
		ACN and water (60 : 40 v/v)					ES = 87		
11	VOR	Develosil C18 column (100 mm × 4.6 mm, 3 μm)	12–100	2.5	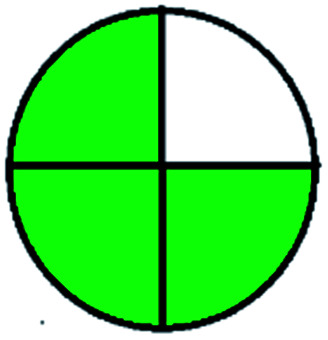	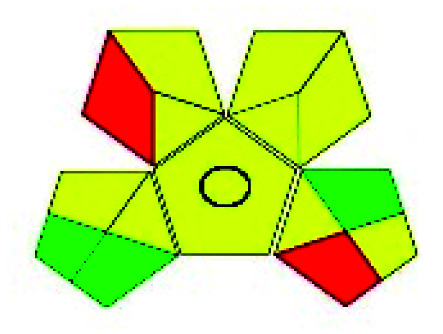	4 + 1 + 3 + 5 = 13	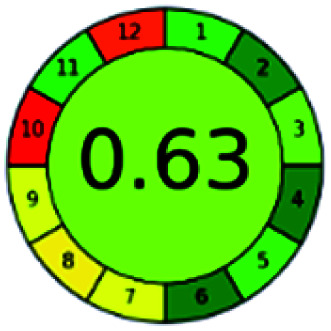	84
		MP A: phosphate buffer 0.05 M (pH 4.5) and ACN (800 : 200% v/v)					ES = 87		
		MP B: CAN and water in the ratio 800 : 200% v/v							
		A : B = 35 : 65							
12	VOR	C18 column (150 mm × 4.6 mm, 5 μm)	1 to 30	4.09	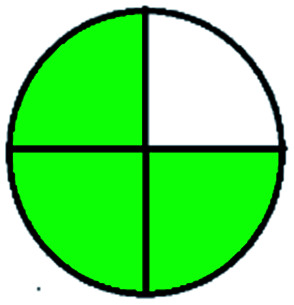	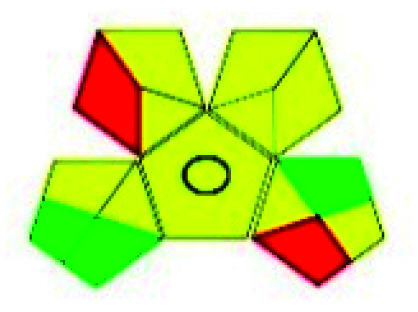	4 + 1 + 3 + 5 = 13	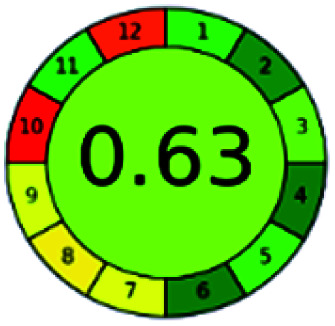	73
		ACN and ultrapure water (50 : 50 v/v)					ES = 87		
13	VOR	Prontosil C-18 (250 mm × 4.6 mm, 5 μm)	5 to 25	7.92	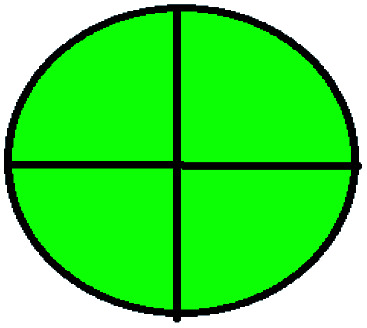	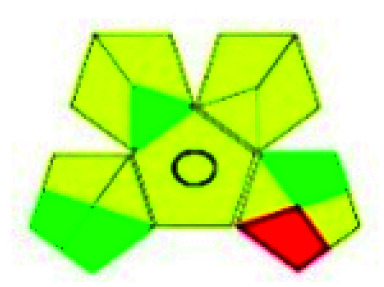	4 + 1 + 3 + 5 = 13	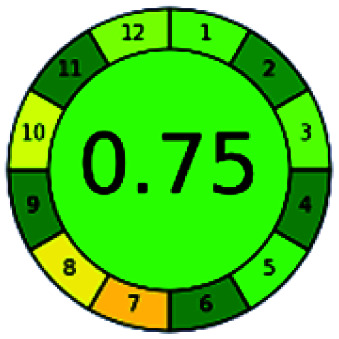	85
		Isopropyl alcohol : water (80 : 20 v/v)					ES = 87		
14	VOR	Inertsil ODS 3V (150 mm × 4.6 mm, 5 μm)	NA	21.78	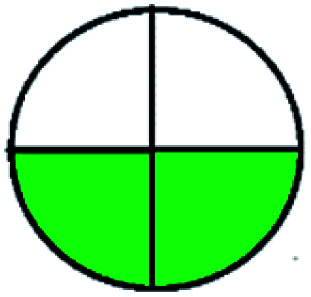	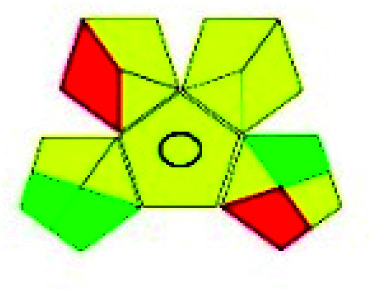	17 + 1 + 3 + 5 = 26	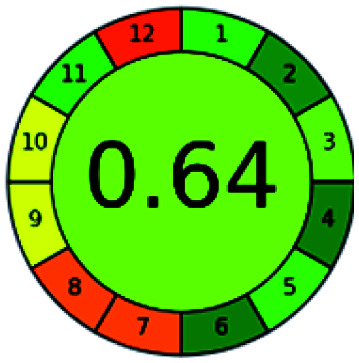	86
		MP A: 0.05 M KH_2_PO_4_ (pH 2.5 buffer)					ES = 74		
		MP B: ACN : MeOH (90 : 10 v/v)							
15	VOR, UFLC	C8 Luna column (250 mm × 4.6 mm, 5 μm)	0.5–50	3.02	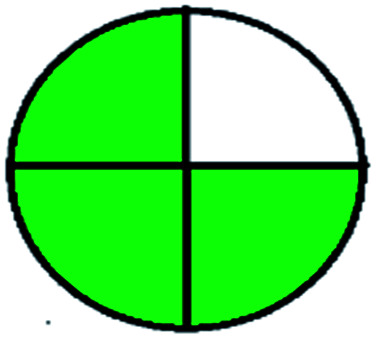	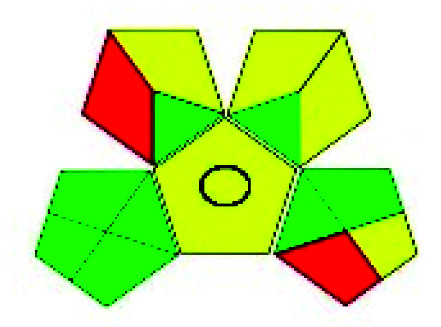	5 + 0 + 3 + 3 = 11	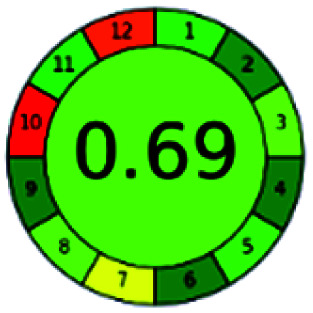	72
		ACN : 0.01% CH_3_COOH (50 : 50 v/v)					ES = 89		

a VOR – voriconazole; MeOH – methanol; ACN – acetonitrile; FeCl_3_ – ferric chloride; KH_2_PO_4_ – potassium dihydrogen phosphate; NaOH – sodium hydroxide; HCl – hydrochloric acid; N – normal; NA – not available; LOQ – limit of quantification; LOD – limit of detection; mg mL^−1^ – microgram per millilitre; MP – mobile phase.

#### 3.3.3. High-performance thin layer chromatography (HPTLC)

HPTLC is a quick, dependable, and accurate qualitative and quantitative drug analysis; it is a viable alternative method for drug testing. Unfortunately, there are just a few HPTLC techniques available for determining VOR alone or in combination.

Khetre *et al.*^87^ utilized HPTLC to develop a technique for detecting VOR in human plasma in API and therapeutic dosage forms. This technique used silica-gel 60 F254 precoated on aluminum sheets as the stationary phase and mobile phase comprising MeOH : toluene (7 : 3 v/v), and VOR is quantified at 255 nm using densitometric analysis. VOR's *R*_f_ values were determined to be 0.58 ± 0.02. The linear connection between the 200–1000 ng per spot concentration range showed an excellent linear regression. The detection and quantification limits were 12.05 and 36.55 ng per spot, correspondingly. This study showed a superb quantification value when compared to the other methods.

Similarly, Dewani *et al.*^88^ developed an HPTLC method to determine VOR in human plasma using the mobile phase combination of triethylamine : MeOH : toluene in the proportion of 0.1 : 4 : 6 v/v/v, in silica gel 60 F254 as a stationary phase. The sample was prepared by dissolving plasma protein precipitation using ACN solvent. The analysis of the VOR has been performed at a wavelength of 254 nm in the concentration range between 50 and 400 ng per band, which exhibits a good range of linearity. The mean rate of drug recovery was determined to be 98.82% for VOR using the reported method.

In another study, Jain *et al.*^89^ quantified VOR in raw materials and cream formulations using the stationary phase of aluminum plates coated using silica-gel 60 RP-18F-254S and mobile phase with a mix of ACN : water (60 : 40 v/v). Under a 200 to 1200 ng per band concentration, the absorbance of 257 nm was calculated with an *R*_f_ of 0.48 ± 0.02. The *R*^2^ value is 0.999, indicating a strong linear correlation. The levels of detection and quantification are 19.99 ng and 60.60 ng, correspondingly. This method helps to identify the VOR in the cream formulation and makes the analysis simplified.

Also, Santosh V. *et al.*^90^ established a method for estimating VOR in pharmaceutical dosage form with a chromatographic separation on precoated aluminum plates using silica gel 60 F254 and mobile phase composed of MeOH : toluene (2 : 8 v/v), tracked at 256 nm by densitometric scanning. The *R*_f_ value of VOR was at 0.45 ± 0.02. The linear range was found to be 400–1600 ng per band. The quantitation and detection limits for VOR were identified to be 61.30 and 20.22 ng per band, correspondingly. The overall assessment of the available reported HPTLC methods is depicted in Table 3.

**Table 3:** Green assessment for the reported HPTLC methods

S. no.	Mobile phase	NEMI	GAPI	AES	AGREE	Ref.
1	MeOH : toluene (7 : 3 v/v)	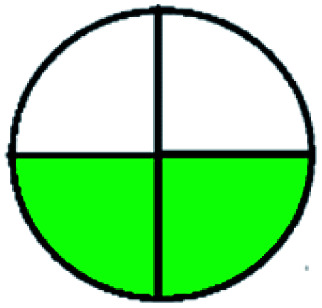	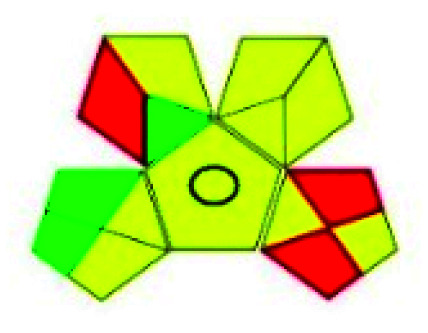	24 + 1 + 3 + 5 = 33	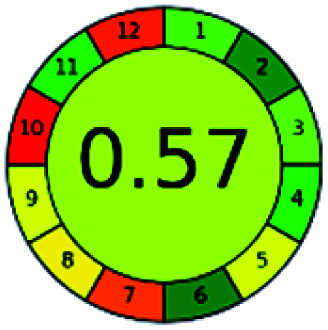	87
				ES = 67		
2	Triethylamine : MeOH : toluene (0.1 : 4 : 6 v/v/v)	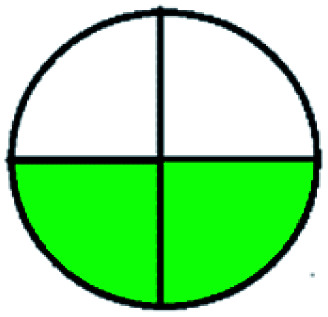	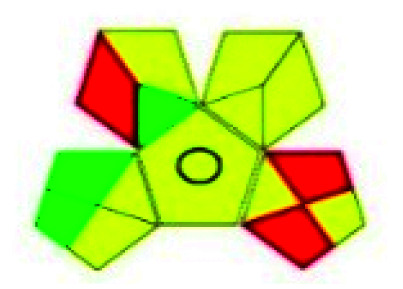	30 + 1 + 3 + 5 = 39	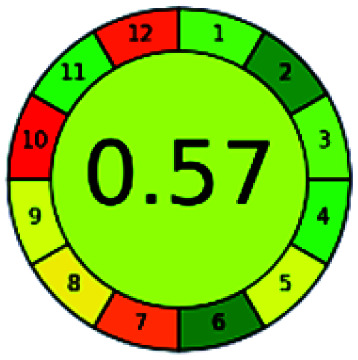	88
				ES = 61		
3	ACN : water (60 : 40 v/v)	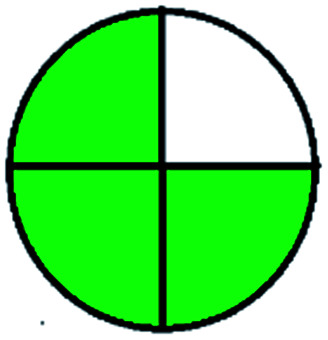	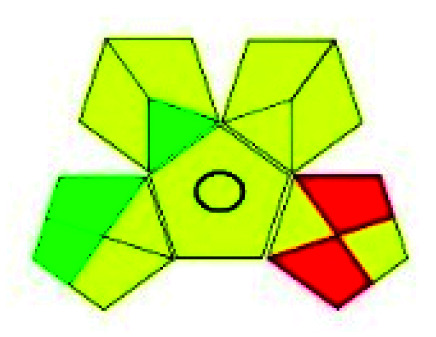	4 + 1 + 3 + 5 = 13	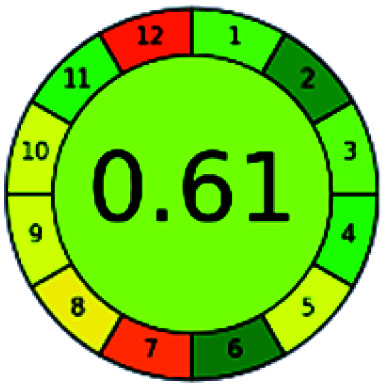	89
				ES = 87		
4	MeOH : toluene (2 : 8 v/v)	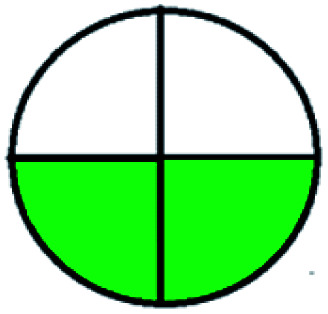	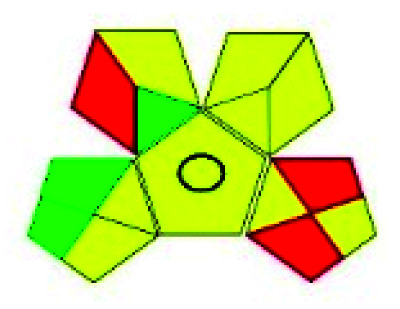	24 + 1 + 3 + 5 = 33	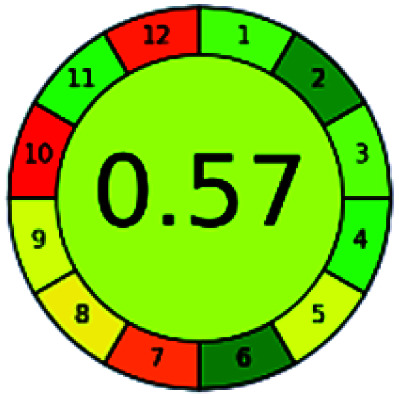	90
				ES = 67		

#### 3.3.4. Bioanalytical methods

Bioanalysis is a critical component of the pharmacokinetic/pharmacodynamic evaluation of a new drug entity, beginning with its discovery and continuing through various stages of drug development and approval. This compilation discusses critical bioanalytical characteristics and their implementation to drug discovery methodologies, which will aid in the production of safer and more effective medications with less time and expense. It is aimed to provide some broad views in this field that will serve as the foundation for a general framework for approaching bioanalysis from the start (*i.e.*, identification of a pioneer chemical) to the different stages of the development process. VOR estimated in various biological matrixes like blood (human), plasma (human, rat, dog, and beagle), serum (human and rat), and aqueous humour (human). Solvent systems like ACN, MeOH, hexane, ethyl acetate, heptane, isoamyl alcohol, hexane, methylene chloride, and diethyl ether were used in different reported methods for the efficient extraction of VOR from various biological matrixes. The minimum linearity concentration reported was 2.49 ng mL^−1^ (ref. 91) to the maximum of 5000 mg L^−1^ (ref. 92). The detection of VOR in biological matrices used different detectors like PDA, UV, fluorescence detection, surface-enhanced Raman spectroscopy, mass spectrometry (MS) and MS/MS. Although each detector has its own advantages, mass spectrometry was the predominantly utilized detector, which has a great potential of more accurate detection even at very low concentration. This also forces the methods to select volatile buffers like formic acid and acetic acid for better compatibility with the organic phase of MeOH and ACN. The bioanalytical techniques for measuring VOR alone and in combination with other medications, along with their greenness assessment, are summarized in Table 4.

**Table 4:** Analytical and greenness assessment data for the reported bioanalytical methods^a^

Drug and instrument	Sample	Extraction	Column	Linearity	NEMI	GAPI	AES	AGREE	Ref.
VOR, multidimensional HPLC with size-exclusion chromatography	Plasma	Sephadex G-25 superfine column of (100 × 10 mm ID)	Spherisorb ODS IMX (250 mm × 4.6 mm, 2.5 μm)	10 to 3000 ng mL^−1^	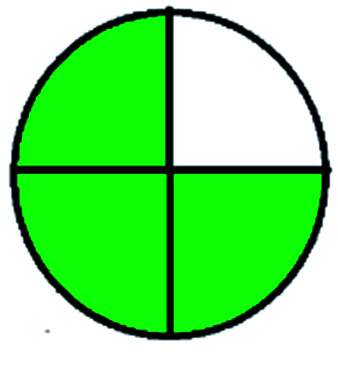	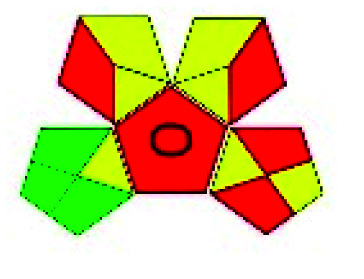	15 + 1 + 3 + 5 = 24	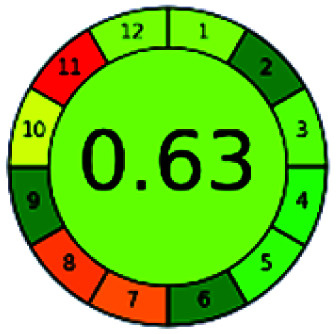	93
			ACN : 0.1 M TEMED phosphate (pH 7.0) (42 : 58 v/v)				ES = 76		
VOR, HPLC	Human plasma	With can	Kromasil C18 (250 mm × 4.6 mm, 5 μm)	0.2–10 μg mL^−1^	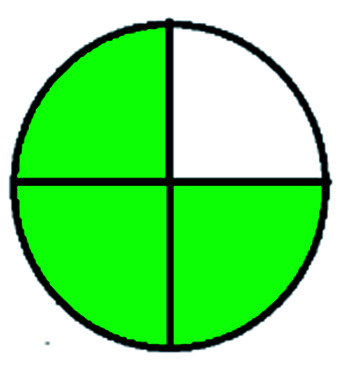	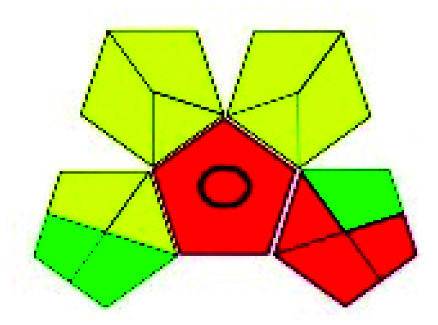	5 + 1 + 3 + 3 = 12	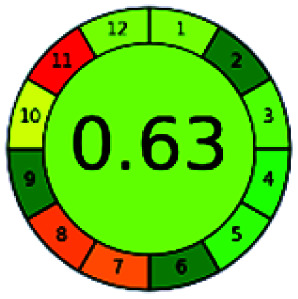	94
			0.04 M ammonium phosphate (pH 6.0 M) : ACN (1 : 1 v/v)				ES = 88		
VOR, HPLC-ESI-MS	Human plasma	Protein precipitation	C18 column (50 mm × 2.1 mm, 3.5 μm)	2.49–293 ng mL^−1^	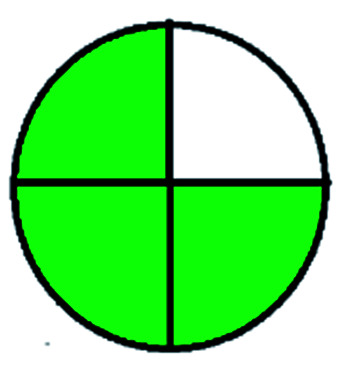	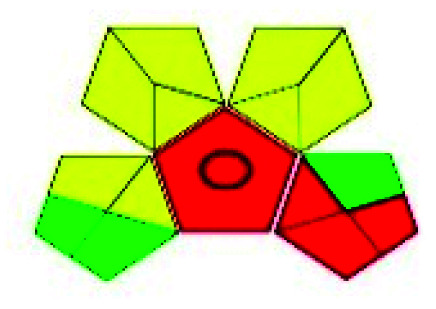	4 + 2 + 3 + 3 = 12	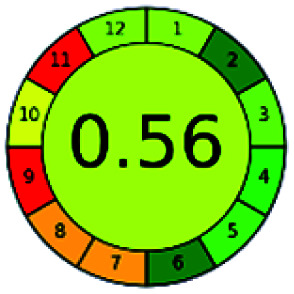	91
			ACN : water (0.1% HCOOH) (40 : 60 v/v)				ES = 88		
VOR, HPLC	Plasma	SPE through Bond Elute columns C18, 100 mg mL^−1^	Luna 5 m C18 column (250 mm × 4.6 mm, 5 μm)	0.2–10 μg mL^−1^	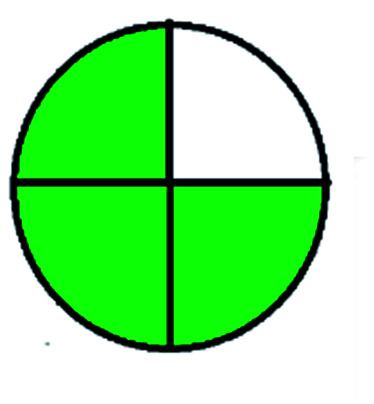	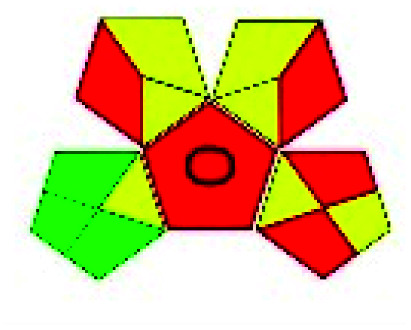	6 + 1 + 3 + 3 = 13	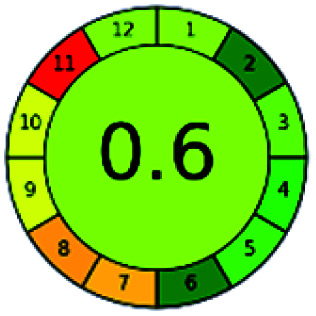	95
			ACN : TEMED (pH 7.4 using phosphoric acid) (45 : 55 v/v)				ES = 87		
VOR, HPLC	Human plasma	Direct injection	Silica particles, bonded with a GFFP (150 mm × 4.6 mm, 5 μm)	0.5–10 μg mL^−1^	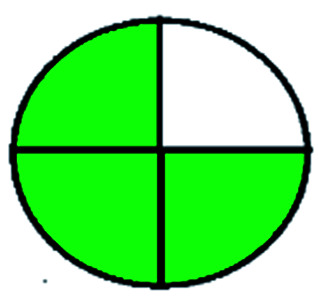	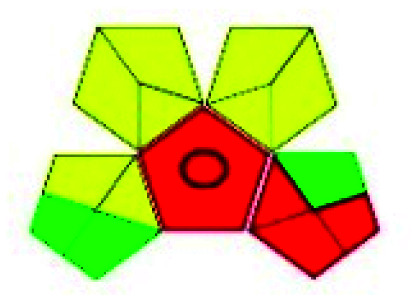	5 + 1 + 3 + 3 = 11	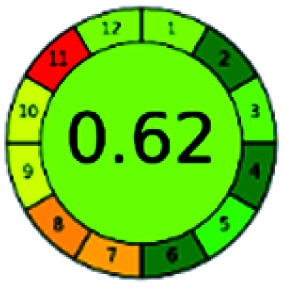	96
			ACN : KH_2_PO_4_ (pH 6.0) (17 : 83 v/v)				ES = 88		
VOR, LCMS	Plasma	Online solid-phase extraction	Waters Oasis HLB (25 mm × 2.0 mm, 2.1 μm)	78–5000 mg L^−1^	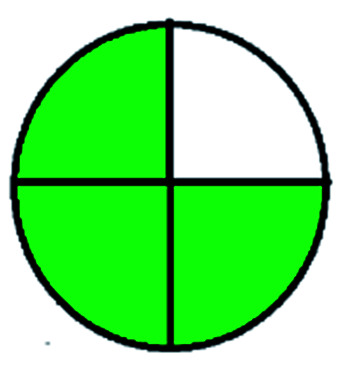	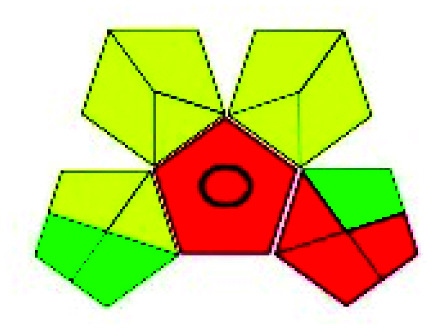	6 + 2 + 3 + 3 = 14	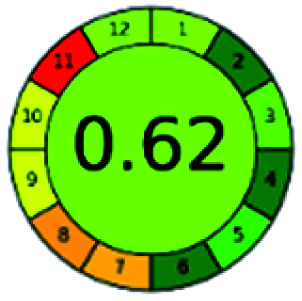	92
		Water/methanol 95 : 5 (v/v)	Water : MeOH (95 : 5 v/v)				ES = 86		
VOR, HPLC	Plasma	LLE	Chromolithic RP 18 monolithic silica rod (100 mm × 4.6 mm)	0.05–10 μg mL^−1^	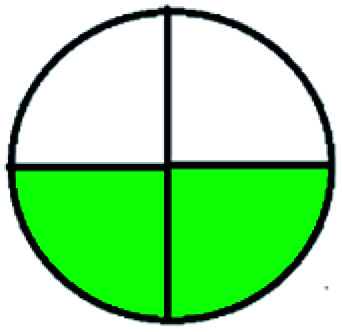	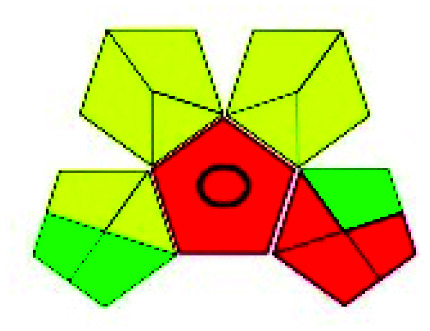	11 + 1 + 3 + 3 = 18	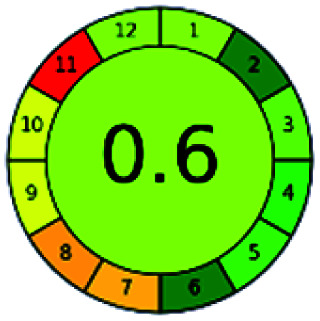	97
			Tetrahydrofuran : ACN : NH_4_HCO_3_ (pH 5.8): (3 : 25 : 72 v/v/v)				ES = 82		
VOR, HPLC-MS	Human plasma	Protein precipitation, automated SPE	LiCrospher 100 RP-18 (125 mm × 4 mm, 5 μm)	78–5000 mg L^−1^	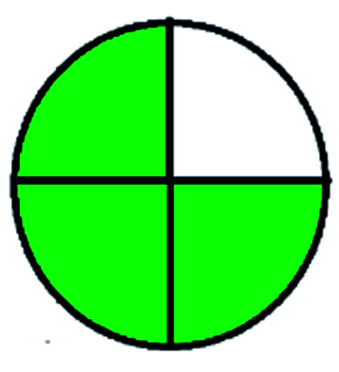	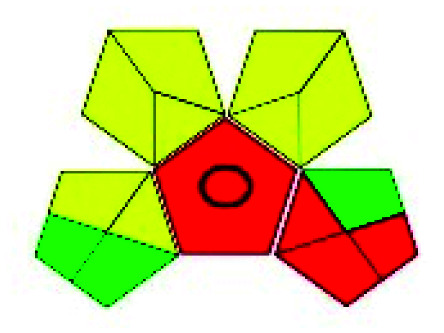	6 + 2 + 3 + 3 = 14	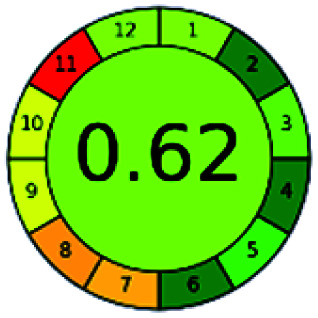	98
			Water : ACN (50 : 50 v/v)				ES = 86		
VOR, LC-MS/MS	Rat plasma	With MeOH	Shim-pack HPLC column (150 mm × 4.6 mm, 5 μm)	50–2500 ng mL^−1^	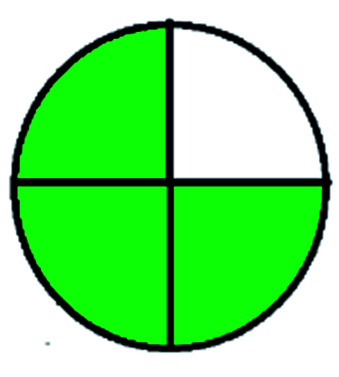	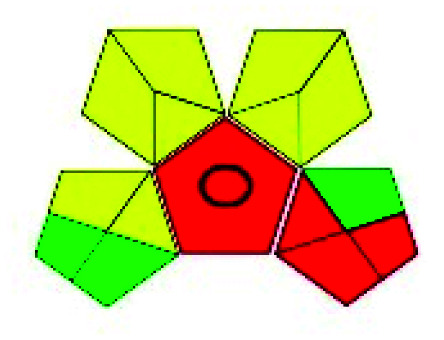	14 + 2 + 3 + 3 = 22	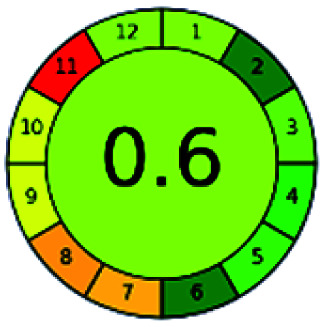	99
			ACN : HCOOH : water (60 : 0.05 : 40 v/v/v)				ES = 78		
VOR, HPLC	Rat and beagle dog plasma	With MeOH : ACN (1 : 2)	Diamonsil C18 (250 mm × 4.6 mm, 5 μm)	0.10–50.0 μg mL^−1^	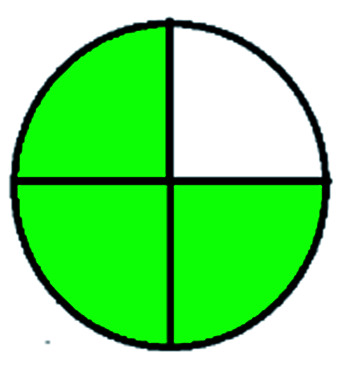	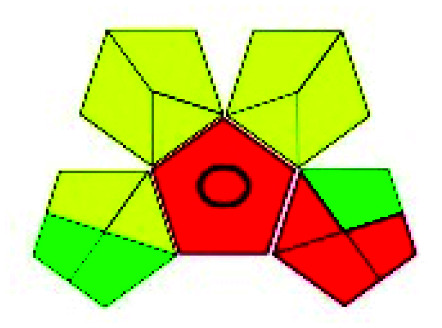	15 + 1 + 3 + 3 = 22	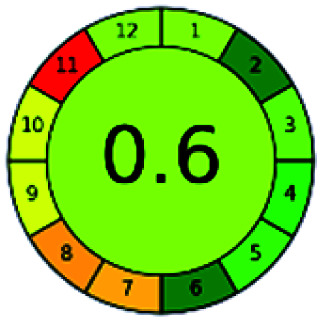	100
			Water : ACN : CH_3_COOH (pH 4.0) (45 : 55 : 0.25 v/v/v)				ES = 78		
VOR, HPLC	Serum	Vortexed with mobile phase	Nova-Pak CN-HP (100 mm × 3.9 mm, 4 μm)	0.4–10.0 mg L^−1^	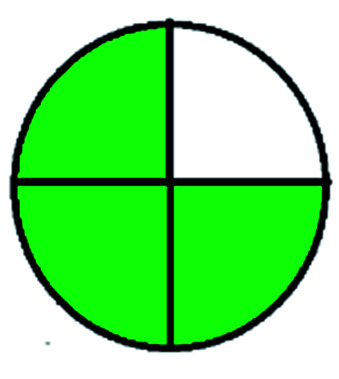	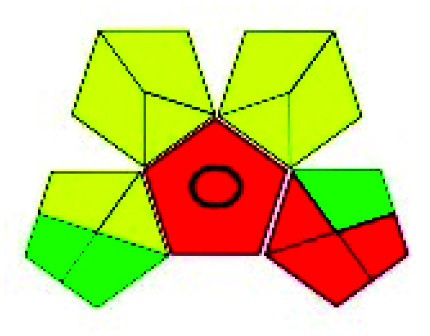	15 + 1 + 3 + 5 = 24	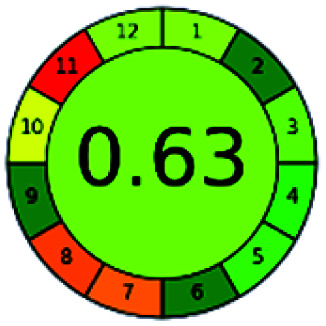	101
			15% ACN (0.1% *n*-butyl amine, 0.2% H_3_PO_4_)				ES = 76		
VOR, HPLC	Plasma	With can	C18 column (150 mm × 4.6 mm)	0.25–16 mg L^−1^	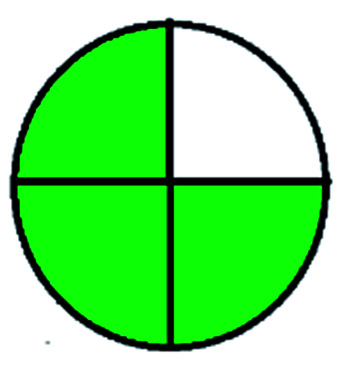	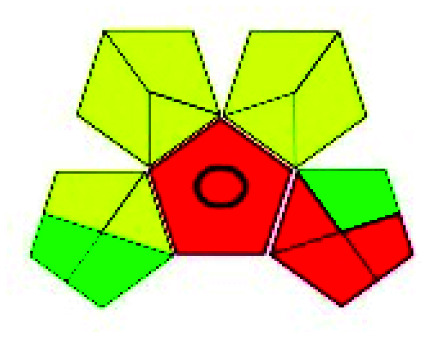	10 + 1 + 3 + 3 = 17	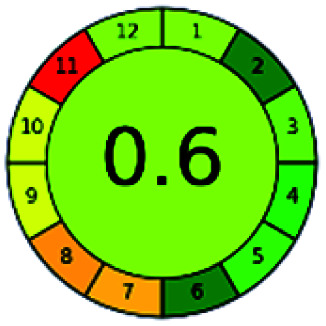	102
			50% MeOH : 0.01 M CH_3_COONa (pH 5.0 v/v)				ES = 83		
VOR, HPLC	Serum and plasma	LLE	Supelcosil LC-18-DB (25 mm × 4.6 mm, 5 μm)	0.1–20 μg mL^−1^	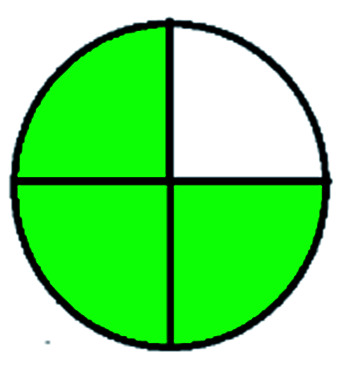	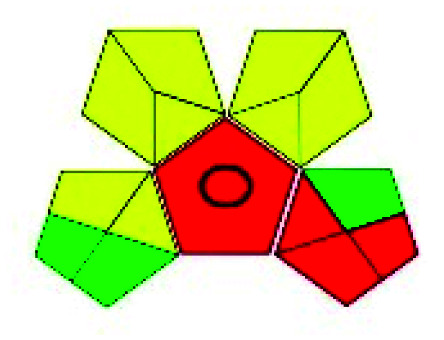	7 + 1 + 3 + 3 = 14	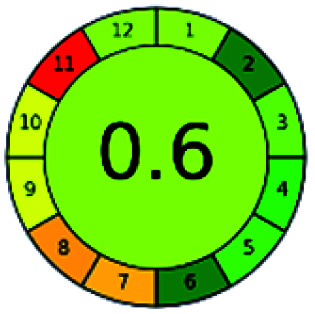	103
			MP A: 10% ACN : 90% 0.01 M K_3_PO_4_ pH 3.0 (v/v)				ES = 86		
			MP B: 100% ACN						
VOR, HPLC-FLD	Human plasma and saliva	With *n*-hexane : ethyl acetate (3 : 1 v/v)	LUNA C18 (250 mm × 3.0 mm, 5 μm)	0.1–10 μg mL^−1^	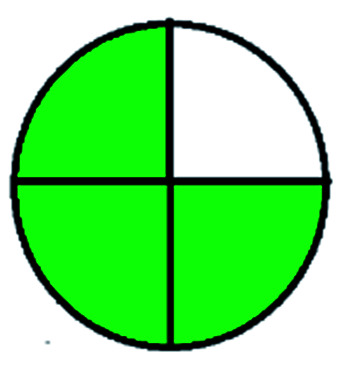	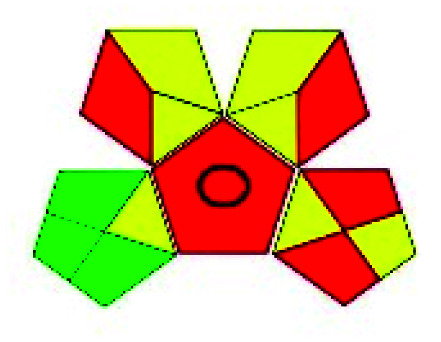	17 + 1 + 3 + 3 = 24	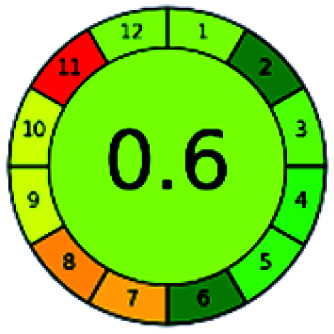	104
			ACN : 0.01 M KH_2_PO_4_ buffer (0.01 M TEMED pH 6.8) (45 : 55 v/v)				ES = 76		
VOR, HPLC	Plasma	With can	LiChrospher-100 RP-18 (125 × 4 mm, 5 μm)	1.5–10 μg mL^−1^	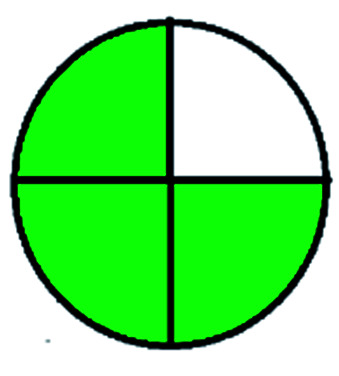	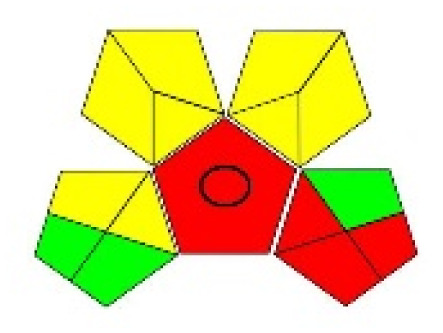	7 + 1 + 3 + 3 = 14	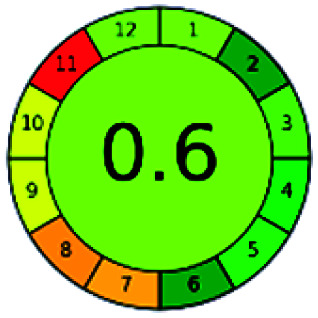	105
			0.04 M ammonium phosphate (pH 6.0) : ACN (60 : 40 v/v)				ES = 86		
VOR, HPLC	Serum	With MeOH	Hibar, LiChrospher C8 RP column (100 mm × 5 mm, 5 μm)	0.26 to 10.1 μg mL^−1^	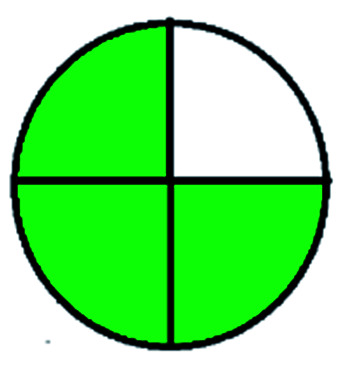	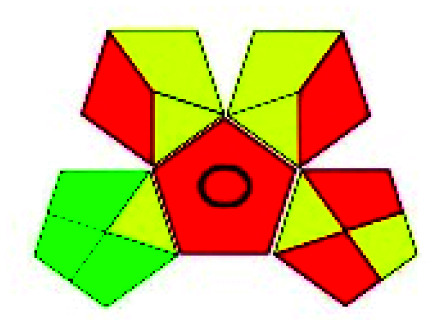	19 + 1 + 3 + 3 = 26	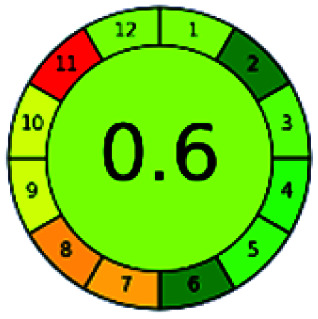	106
			0.04 M KNaPO_4_ (pH 6.0) : water : ACN (45 : 2.5 : 52.5 v/v/v)				ES = 74		
VOR, LCEIMS	Aqueous humour	NA	C18 column (300 mm × 5 mm, 15 μm)	0.02–30 μg mL^−1^	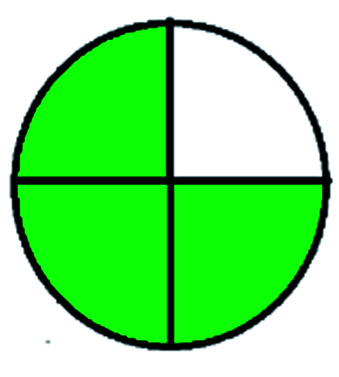	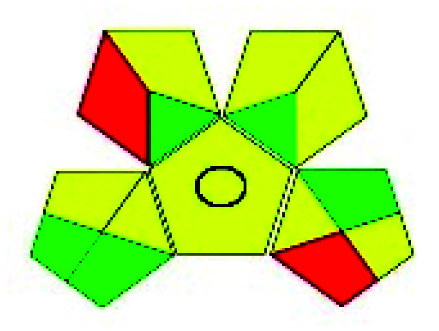	15 + 2 + 3 + 3 = 23	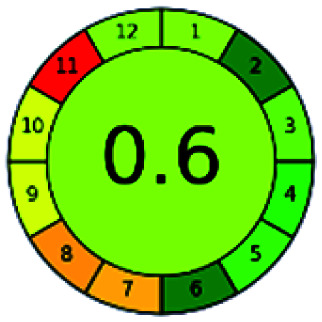	107
			70% ACN : 30% water : 0.01% TFA				ES = 77		
VOR, HPLC-ESI-MS	Plasma	Protein precipitation	C18 column (50 mm × 2.1 mm, 3.5 μm)	2.49–293 ng mL^−1^	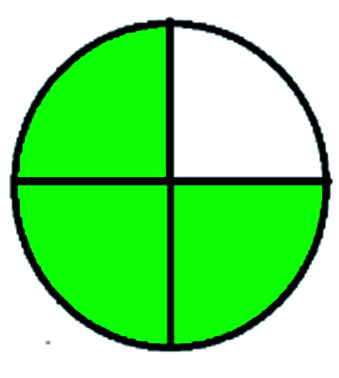	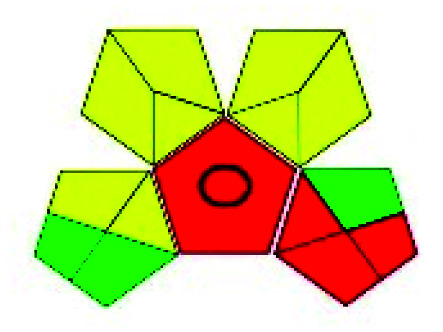	10 + 2 + 3 + 3 = 18	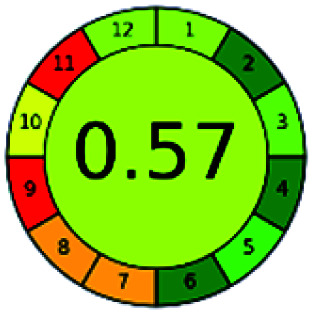	108
			ACN : water (0.1% HCOOH) 40 : 60 v/v				ES = 82		
VOR, LC-MS/MS	Serum	Vortexed and then centrifuged	C18 column (100 mm × 3.0 mm, 2.6 μm)	0.1 and 10.0 μg mL^−1^	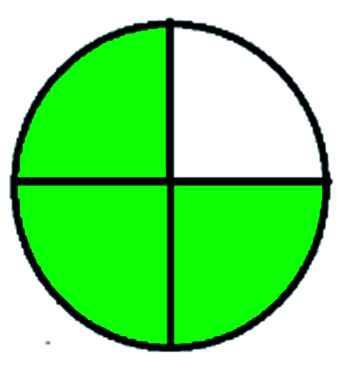	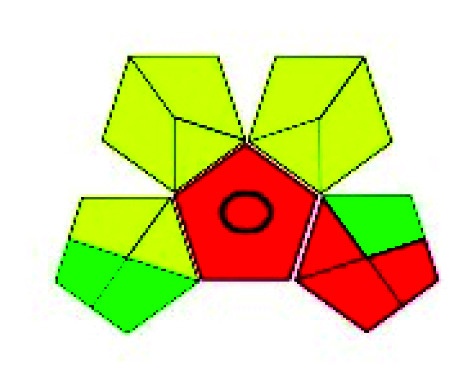	7 + 2 + 3 + 3 = 15	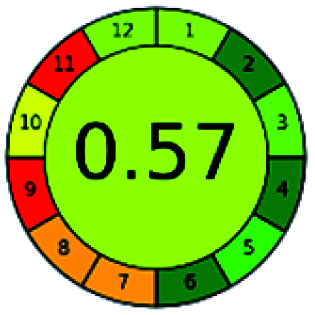	109
			MP A: 0.1% HCOOH : 2 mM CH_3_COONH_4_				ES = 85		
			MP B: 2 mM CH_3_COONH_4_, 0.1% HCOOH in MeOH						
VOR *N*-oxide VOR, LCMS	Bovine serum	Centrifugation	HyPURITY Aquastar C18 (50 mm × 2.1 mm, 5 μm)	0.10–10.08 mg L^−1^	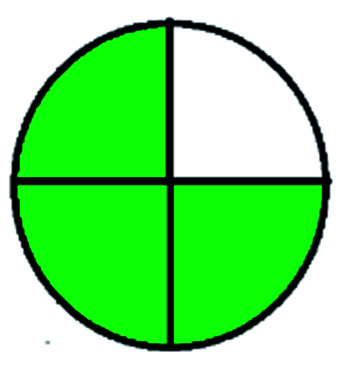	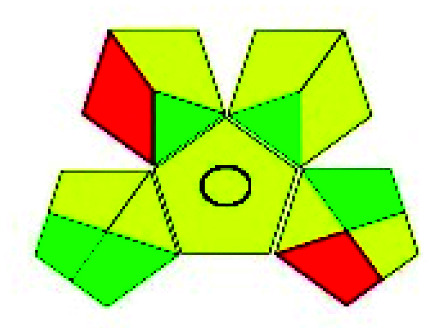	7 + 1 + 3 + 3 = 14	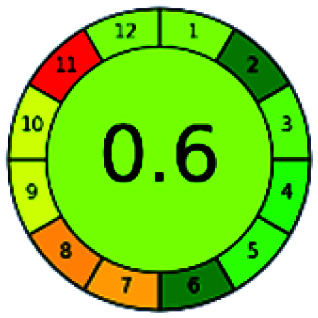	110
			MP A: CH_3_COOH, CH_3_COONH_4_ and TFA				ES = 86		
			MP B: water : MeOH						
VOR, HPLC	Human serum	With ice-cold ACN	SunFire C18 (150 mm × 4.6 mm, 5 μm)	0.25–16.0 μg mL^−1^	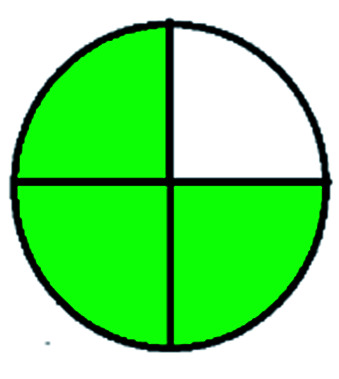	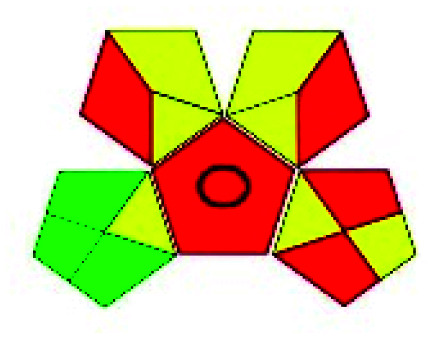	6 + 1 + 3 + 3 = 13	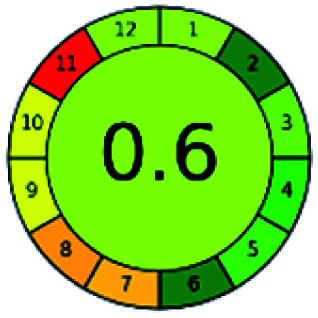	111
			ACN : ultrapure water (70 : 30 v/v)				ES = 87		
VOR, LC-MS-MS	Human serum	Protein precipitated with 200 μL of ACN	EC-C18 (50 mm × 3.0 mm, 2.7 μm)	0.05–10 μg mL^−1^	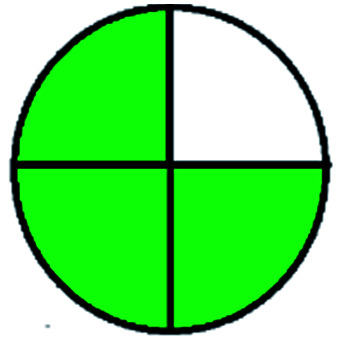	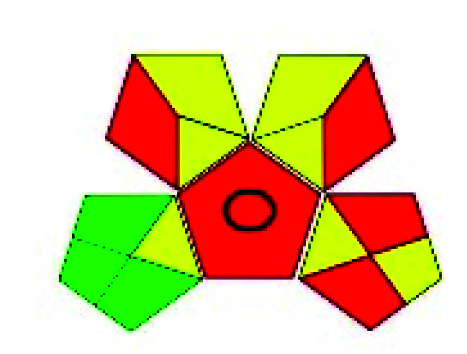	5 + 1 + 3 + 3 = 12	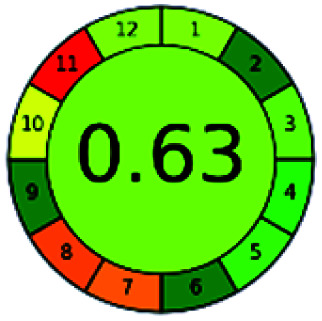	112
			ACN : 0.1% HCOOH in 10 mM CH_3_COONH_4_ (50 : 50 v/v)				ES = 88		
VOR, HPLC-FLD	Human plasma and serum	Protein precipitation and ACN extraction	ODS HYPERSIL column (250 mm × 4.6 mm, 5 μm)	0.1–10 μg mL^−1^	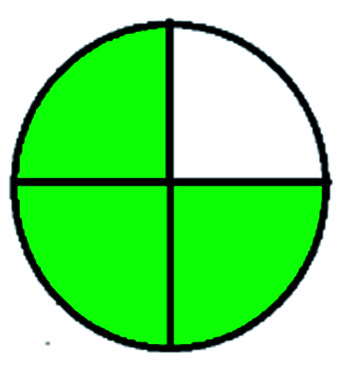	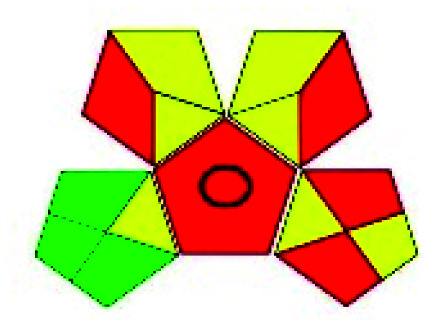	5 + 1 + 3 + 3 = 12	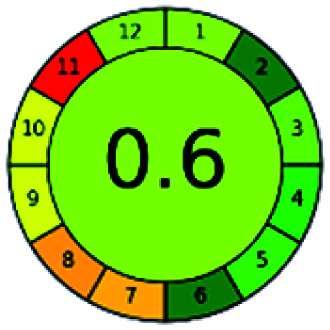	113
			0.1 M CH_3_COONH_4_ solution, ACN and TFA (409 : 590 : 1 v/v/v)				ES = 88		
VOR, HPLC	Human blood	With hexane and ethyl acetate	Eclipse X DB C18 (4.5 mm × 5 μm)	1.0 to 8.0 μg mL^−1^	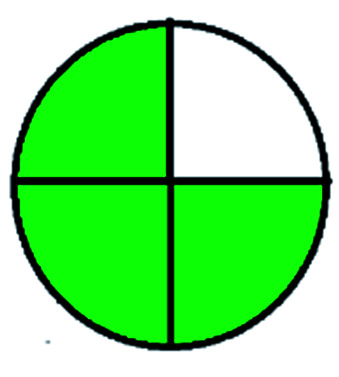	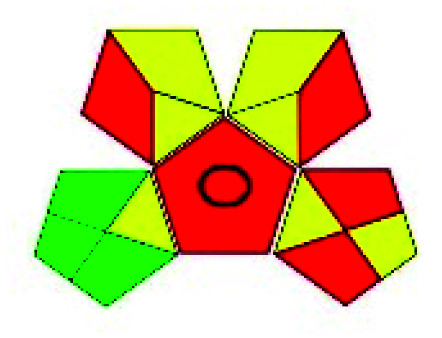	16 + 1 + 3 + 3 = 23	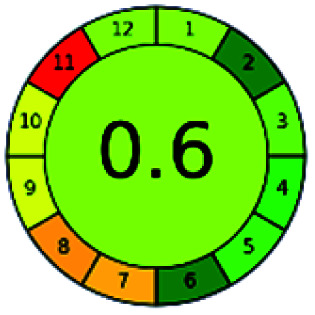	114
			ACN : water (50 : 50 v/v)				ES = 77		
VOR, HPLC-FLD	Human serum	Protein precipitation with ACN	LiChrospher RP-18e column (125 mm × 4 mm, 5 μm)	VOR 0.2–20.0 μg mL^−1^	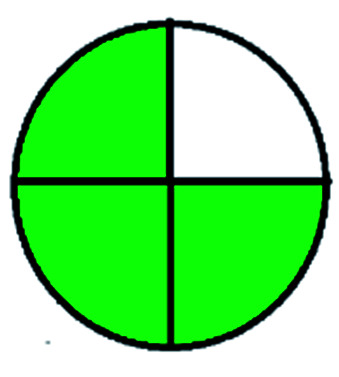	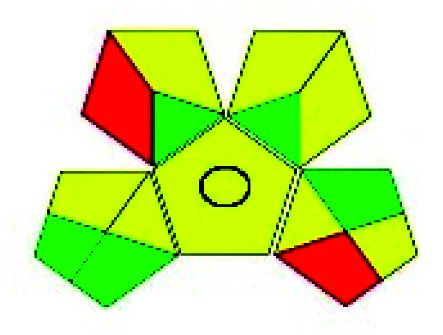	13 + 1 + 3 + 3 = 20	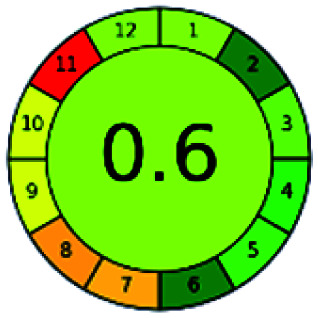	115
			10 mM KH_2_PO_4_ (10 mM of TEMED pH 6.5) : ACN 65 : 35 (v/v)				ES = 80		
VOR, HPLC	Plasma	Ultrafiltration method with tween 80	LiChrospher (125 mm × 4 mm, 5 μm)	0.05–10.0 μg mL^−1^	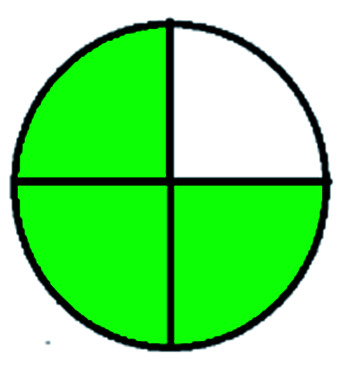	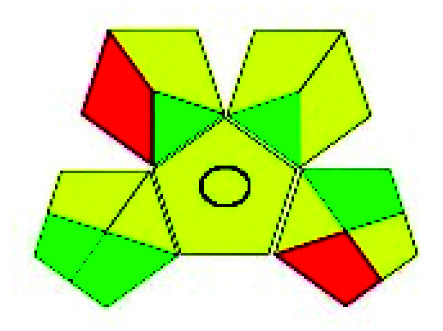	15 + 1 + 3 + 3 = 22	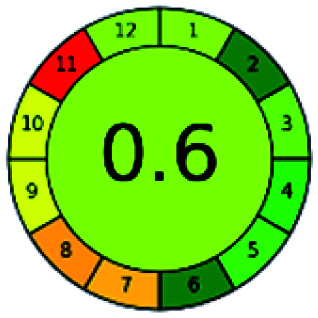	115
			ACN : 10 mM KH_2_PO_4_ (10 mM of TEMED) (pH 6.5) (35 : 65 v/v)				ES = 78		
VOR, LCMS	Human plasma	Vortexed and centrifuged by using MeOH	C18 column (100 mm × 2.1 mm × 3.5 μm)	0.1–10.0 μg mL^−1^	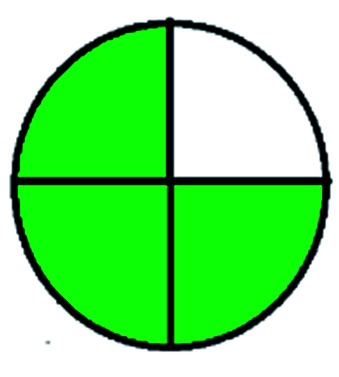	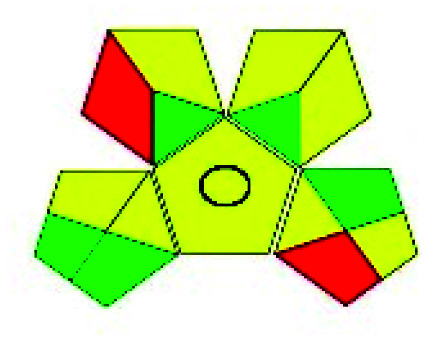	15 + 1 + 3 + 5 = 24	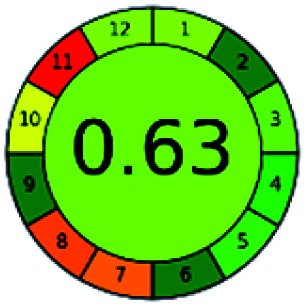	116
			MeOH : 0.1% HCOOH (70 : 30 v/v)				ES = 76		
VOR, surface-enhanced Raman spectroscopy	Plasma	Centrifugation	Diamonsil C18	0.41–6.12 μg mL^−1^	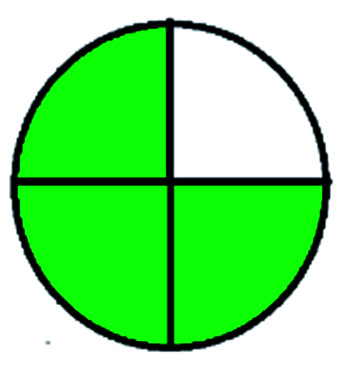	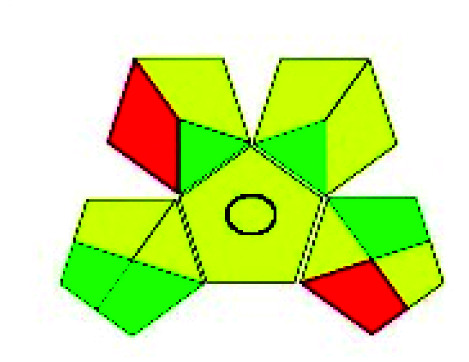	4 + 1 + 3 + 3 = 11	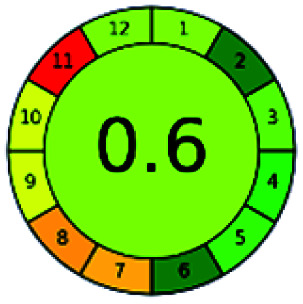	117
			ACN : 0.1% HCOOH (43 : 57 v/v)				ES = 89		
VOR, HPLC	Humans	Centrifugation	C18 column (250 mm × 4.6 mm, 3.5 μm)	0.125–10 μg mL^−1^	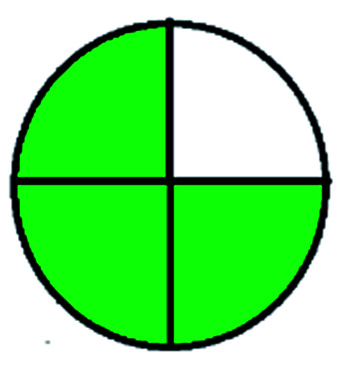	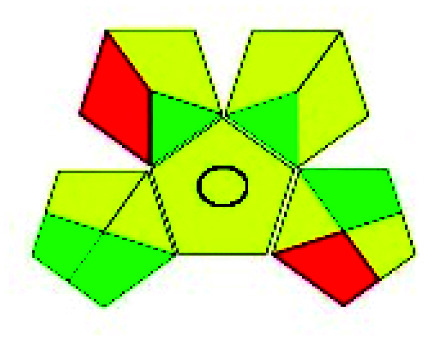	5 + 1 + 3 + 3 = 12	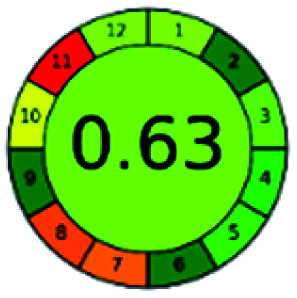	118
			ACN : 0.05 M CH_3_COONH_4_ : MeOH (20 : 40 : 40 v/v/v)				ES = 88		
VOR, HPLC	Beagle plasma	Vortexed and centrifuged by using MeOH	Venusil XBP C18 (250 mm × 4.6 mm, 5 μm)	200–100 000 ng mL^−1^	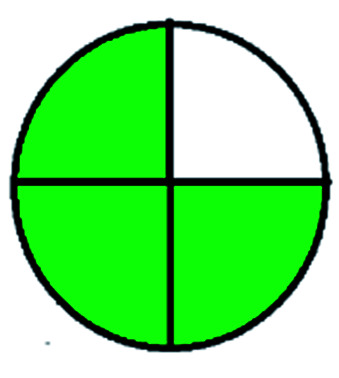	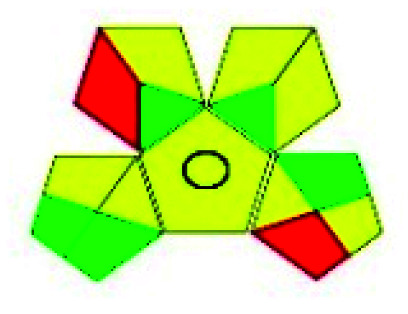	15 + 1 + 3 + 3 = 22	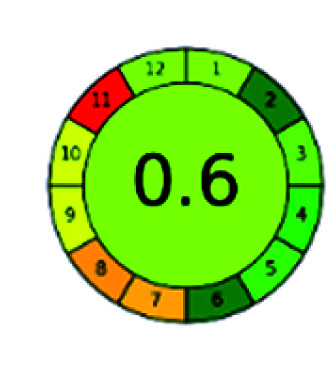	119
			ACN : 20 mM KH_2_PO_4_ (65 : 35 v/v)				ES = 78		
VOR, ITC, HPLC	Human serum	Heptane–isoamyl alcohol (90 : 10 v/v)	Zorbax SB-C18 (250 mm × 4.6 mm, 5 μm)	0.5–5.0 μg mL^−1^	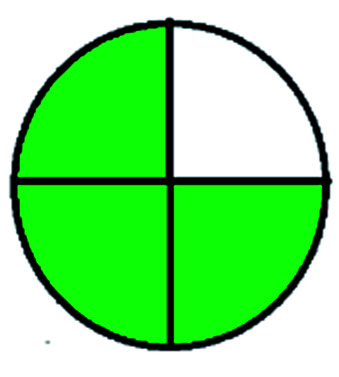	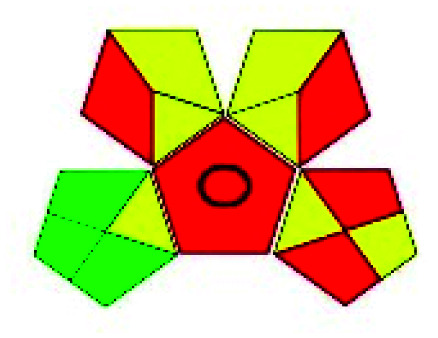	19 + 1 + 3 + 3 = 26	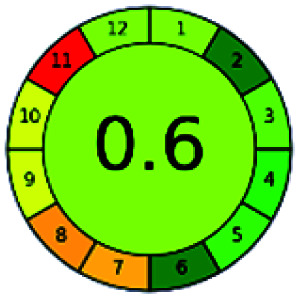	120
			50 mM phosphate buffer (pH 6.0 with 1 M KOH) : MeOH : ACN (35 : 20 : 45) (v/v/v)				ES = 74		
VOR, PSC, HPLC	Human plasma	Hexane–methylene chloride (70 : 30 v/v)	C8 plus (250 mm × 3 mm, 5 μm)	VOR 0.2–10.0 mg L^−1^	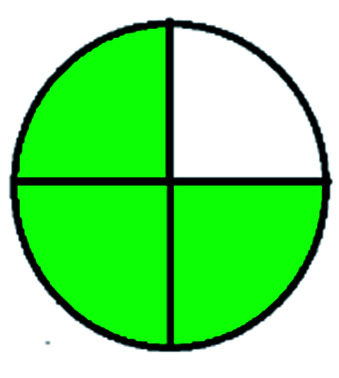	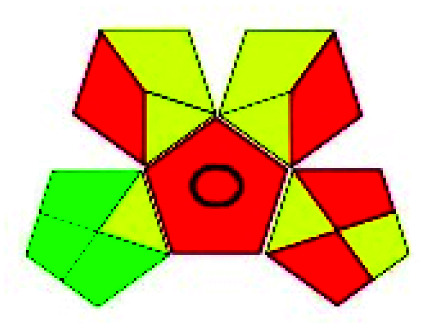	15 + 1 + 3 + 3 = 22	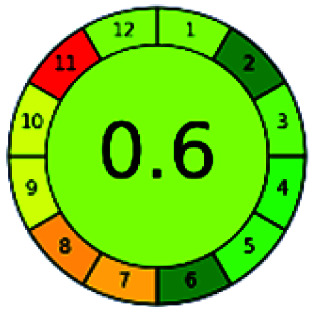	121
			Water : 0.04 M NaKHPO_4_ : ACN (2.5 : 45 : 52.5 v/v/v)	PSC 0.05–10.0 mg L^−1^			ES = 78		
VOR, PSC, HPLC	Human plasma	LLE with diethyl ether	ReproSil-Pur Basic C18 (150 mm × 2 mm × 5 μm)	1.0–20.0 μg mL^−1^	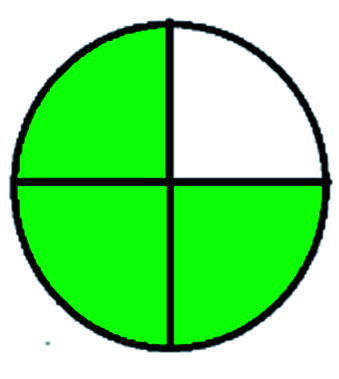	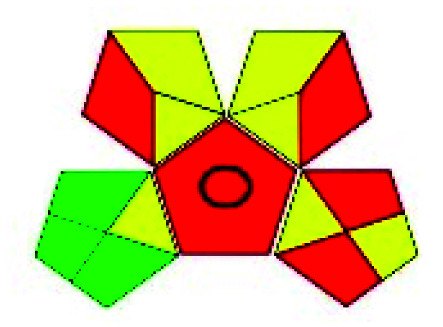	7 + 1 + 3 + 3 = 14	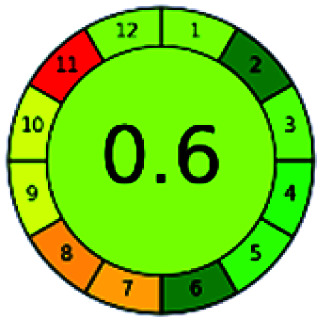	122
			ACN : 0.09 M monobasic ammonium phosphate (pH 5.3) (50 : 50 v/v)				ES = 86		
VOR, ITC, PSC, HPLC-MS	Human plasma	Protein precipitation extraction with ACN	C18 Atlantis T-3 (150 mm × 4.6 mm, 5 μm)	ITC 0.04–1.18 μg mL^−1^	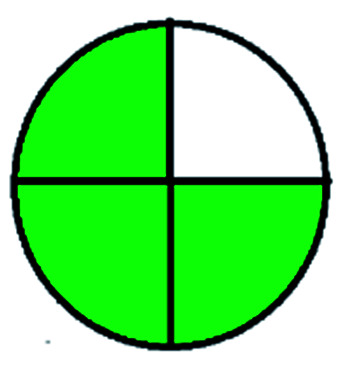	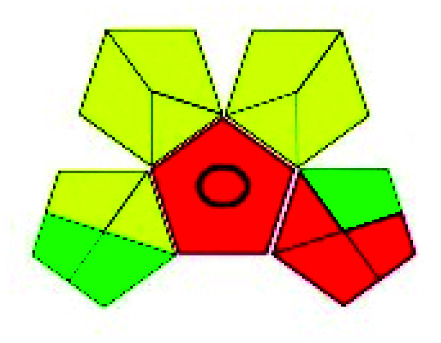	6 + 1 + 3 + 3 = 13	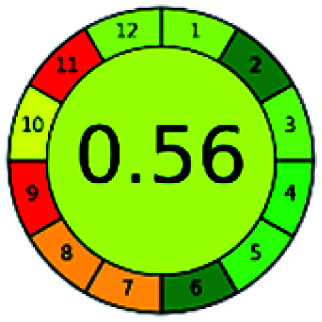	123
			Water (0.05% HCOOH) : ACN 50 : 50 and 20 : 80 from 6.5 min	PSC 0.04–3.20 μg mL^−1^			ES = 87		
				VOR 0.09–8.32 μg mL^−1^					
VOR, PSC, FLZ, ITZ, LC-MS/MS	Human serum	Protein precipitation	Phenomenex Luna C8 (50 mm × 2 mm, 3 μm)	VOR 0.01–10 μg mL^−1^	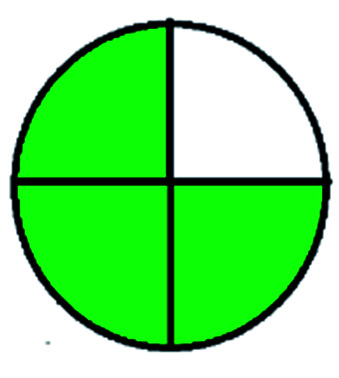	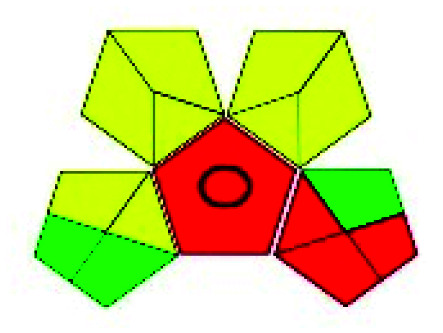	5 + 1 + 3 + 3 = 11	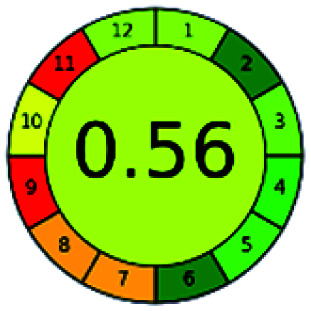	124
			MP A: 0.1% HCOOH and 10 mM HCOONH_4_ in water	POS 0.02–40 μg mL^−1^			ES = 89		
			MP B: 0.1% HCOOH in ACN	FLU 0.2–200 μg mL^−1^					
				ITZ 0.02–20 μg mL^−1^					
VOR, TFL, HPLC	Plasma	Without extraction	Shim-Pack XR-ODS (100 mm × 2.0 mm, 2.2 μm)	0.1 μg mL^−1^ and 0.5 μg mL^−1^	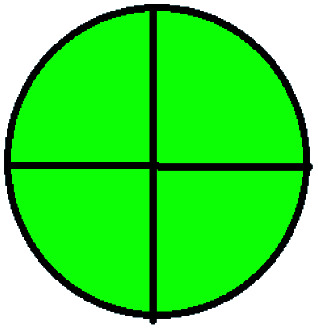	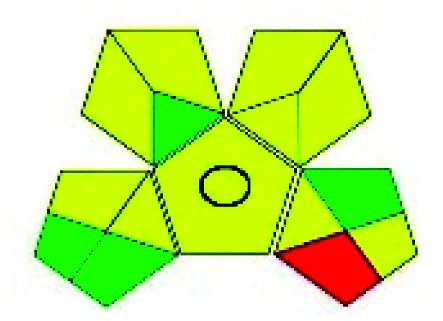	4 + 1 + 0 + 1 = 6	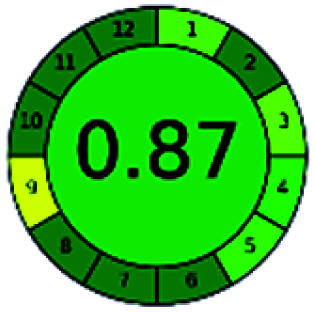	125
			50 mM phosphate buffer (pH 3.0)/propylene carbonate : ethanol (10 : 90 v/v)				ES = 94		
VOR, ITC, PSC, UPLC-MS/MS	Human plasma	Centrifugation	BEH C18 column (50 mm × 2.1 mm, 1.7 μm)	VOR 0.13 and 6.54 mg L^−1^	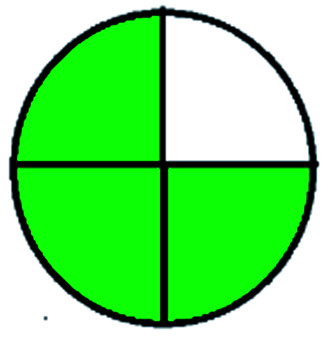	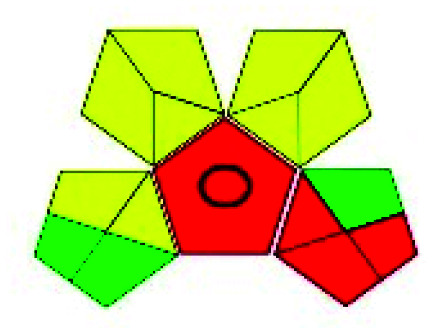	7 + 1 + 3 + 3 = 14	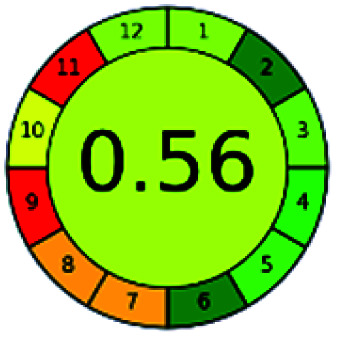	126
			MP A: 10 mM CH_3_COONH_4_ in water and 0.1% HCOOH	POS 0.16 and 5.66 mg L^−1^			ES = 86		
			MP B: MeOH and 0.1% HCOOH	ITC 0.18 and 3.64 mg L^−1^					
VOR, IMB, UPLC-MS/MS	Rat plasma	Protein precipitation by can	Acquity UPLC BEH C18 column (2.1 mm × 50 mm, 1.7 μm)	Imatinib, *y* = (2.70755*x* + 166.088, *r*^2^ = 0.99818)	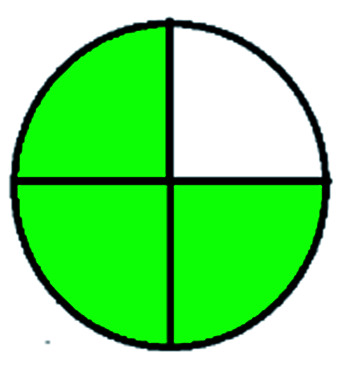	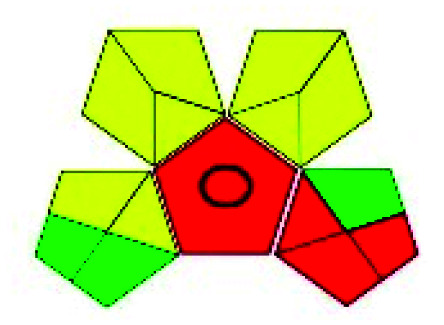	6 + 1 + 3 + 3 = 13	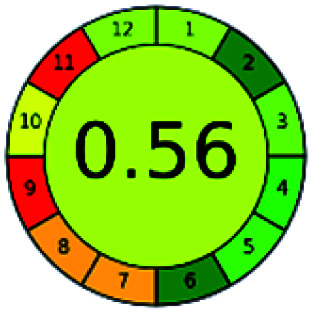	127
			ACN : 0.1% HCOOH in water (50 : 50 v/v)	VOR, *y* = (5.20704*x* + 3.45498, *r*^2^ = 0.99939)			ES = 87		

a VOR – voriconazole; HPLC – high-performance liquid chromatography; *R*_*t*_ – retention time; SPE – solid-phase extraction; HCOOH – formic acid; KH_2_PO_4_ – potassium dihydrogen phosphate; NaH_2_PO4 – sodium dihydrogen phosphate; DAP – daptomycin; MeOH – methanol; ACN – acetonitrile; ITC – itraconazole; PSC – posaconazole; TED – tedizolid; LLE – liquid–liquid extraction; FLZ – fluconazole; IMB – imatinib; TFL – tadalafil; LC-MS/MS – liquid chromatography-tandem mass spectrometry; TEMED – *N*,*N*,*N*,*N*-tetramethyl ethylene diamine; K_3_PO_4_ – potassium phosphate; HPLC-FLD high-performance liquid chromatography fluorescence detection; GPPP – glycine phenylalanine–phenylalanine peptide.

#### 3.3.5. Miscellaneous methods

A chromatographic method was proposed by Babu *et al.*^128^ for analyzing VOR in pharmaceutical formulations using a quality by design approach. The stationary phase utilized for the method was the C18 column (250 mm × 4.6 mm, 5 μm), and a 50 : 50 v/v blend of ACN and water as the mobile phase with a 1.0 mL min^−1^ flow rate. Three variables were taken into account while determining robustness, which were the proportion of ACN in the mobile phase, the pH, and the flow rate; a rising inflow leads to a reduction in the concentration of the drug detection, while the proportion of ACN and the pH had significantly less impact on the response. A correlation coefficient of 0.9999 was determined to prove the noteworthiness of the developed method. The RSD result (0.45%, *n* = 24) showed that the analytical technique is precise and accurate and shall be used for long-term use due to applying the analytical quality by design concept.

In another study, Lin *et al.*^129^ established a technique for determining VOR concentrations in a patient's plasma sample using sweeping-micellar electrokinetic chromatography on a fused silica capillary of 75 cm × 50 mm ID column. The solution included 110 mM sodium dodecyl sulfate, 20% ACN, and 40 mM phosphoric acid. The voltage applied was −23 kV, and the wavelength of detection was 254 nm. VOR was isolated from endogenous materials within 10.5 min under optimum analytical conditions, limiting the detection at 0.075 g mL^−1^. Plasma VOR levels were quantified in 16 individuals; the findings were consistent with those acquired by the HPLC method. This method may be recognized as a new technique by applying a new concept called sweeping-micellar electrokinetic chromatography. Still, this technique was eventually used to develop a green analytical method. In this technique, the authors used ACN as an organic modifier, making this method vulnerable towards eco-friendly usage. However, these types of methods are encouraged and need to be optimized by applying some biodegradable solvents.

Similarly, Corbini *et al.*^130^ devised a new technique for quantifying VOR using differential pulse polarography (DPP) in pharmaceuticals. A distinct peak (−1.01 V *versus* Ag/AgCl) was produced using a 0.01 M KH_2_PO_4_ buffer (pH 4.5) supporting electrolyte. Accordingly, the concentration stood linear in the series of 0.5 to 5.0 μg mL^−1^, through a LOD and LOQ of 0.03 and 0.10 μg mL^−1^. This resultant method consumes fewer toxic substances and may be used for sustainable development.

Smith *et al.*^131^ developed a GCMS method to determine VOR in serum. The sample extraction was performed with the help of cold methanol and ethyl acetate by adding the internal standard THC-deuterium 9 (THC-d9) and derivatized using *N*,*O*-bis(trimethylsilyl)trifluoro acetamide (BSTFA). The run time used for every run was about 11 min with a linearity range of 0.4 to 10 μg mL^−1^. This method showed a better result with no interaction with the other drugs. It was the best adaptable one for VOR analysis in the serum without interference from the other substances.

Recently, Lerch *et al.*^132^ developed a rapid and efficient analytical technique called paper spray mass spectrometry (PSMS), used for the first time to quantify VOR in the complex biological matrix without using chromatographic or traditional sample separation. An innovative PSMS technique for quantitating VOR in equine tears has been determined and corroborated over a series of 10 to 1000 ng mL^−1^. The method demonstrates excellent accuracy, linearity (*r*^2^ > 0.990), inter and intra-day precision, and selectivity for the quantitation limit in equine tears. VOR was computed using three products compared to an internal standard with an isotope label, voriconazole-d3, with a 250 ng mL^−1^ standard concentration in samples. The authors further applied this technique to the analysis of 126 test samples, and acquired the sample dilution's integrity, and carryover impact was further examined and determined within acceptable limits.

In another study, Sahitya *et al.*^133^ used *Candida albicans* as the test microorganism to develop a novel microbiological technique for examining VOR tablets. It was necessary to experiment with different mediums, species, and circumstances to optimize the diffusion test. During a prospective validation, the method showed excellent linearity (0.995), accuracy less than 2% RSD, and consistency (mean recovery = 101.77%). VOR was evaluated using HPLC, which was used as a comparative method for the study. The results of both the microbiological and HPLC techniques have been compared using the Student's *t*-test. The VOR content measured from both ways has demonstrated a high degree of consistency. When employed in dosage forms for regular quality control analysis of VOR, the newly developed microbiological analytical technique gives a genuine indicator of biological activity. It may be utilized to detect actual biological activity.

Kaur *et al.*^134^ recently devised and evaluated an RP-HPLC technique for determining VOR using an AQbD approach. The authors used a Taguchi design to address specific constraints that affect factors, including theoretical plate count, retention time, peak area, and peak tailing. Response surface design is used for optimization studies to identify critical constraints such as organic phase mix in the mobile phase and flow rate that affect variables such as peak tailing, theoretical plates, peak area, and retention time by a central composite design. The optimum operating conditions for the technique were determined using graphical refinement and then verified by Monte Carlo simulations. The optimum mobile phase condition was ACN and 0.05% acetic acid (pH 4) (50 : 50 v/v). The flow rate of 1 mL min^−1^, at 256 nm detection, demonstrated linearity within 0.1–50 μg mL^−1^ in Hanks balanced salt solution and methanol. Corroboration data showed the proposed analytical method's efficacy and sensitivity in quantifying VOR. Quantification of VOR in pharmaceutical nano-formulations was effectively accomplished using the established analytical technique. The above-mentioned miscellaneous methods were assessed using the four green assessment tools and are depicted in Table 5.

**Table 5:** Green assessment for the reported miscellaneous methods

S. no.	Method and solvent/chemicals	NEMI	GAPI	AES	AGREE	Ref.
1	HPLC AQbD	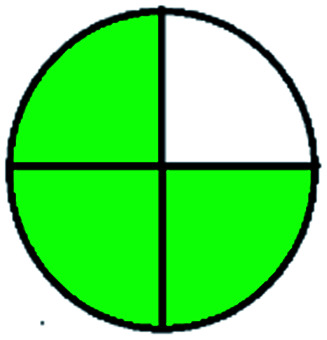	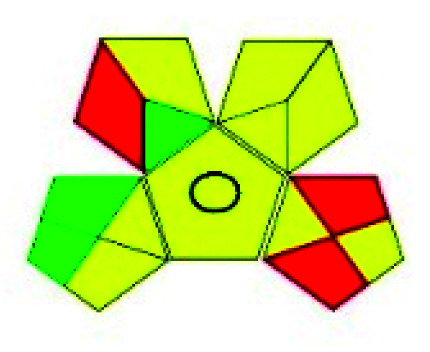	8 + 1 + 3 + 5 = 17	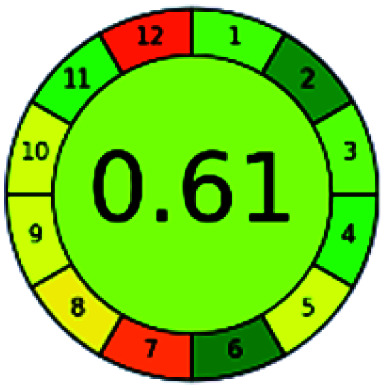	128
	50 : 50 v/v ACN and water			ES = 83		
2	Sweeping-micellar electrokinetic chromatography	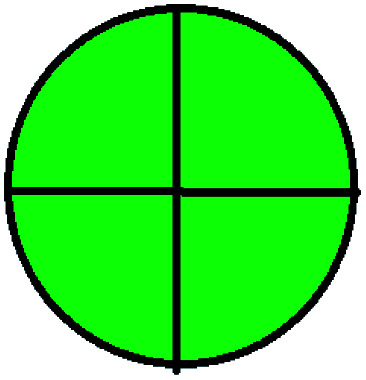	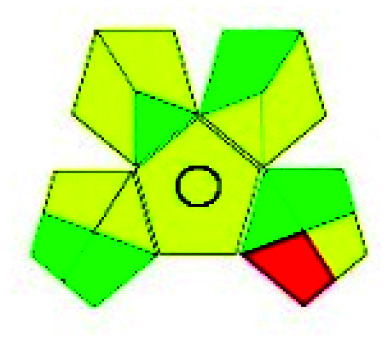	2 + 1 + 0 + 0 = 3	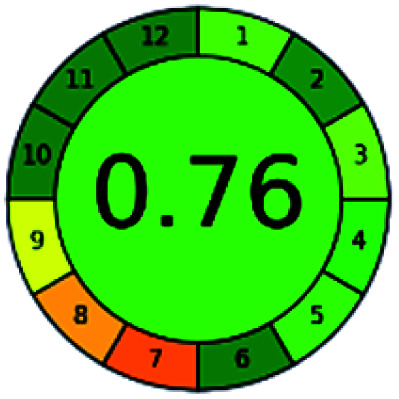	88
	110 mM sodium dodecyl sulfate, 20% ACN, and 40 mM phosphoric acid			ES = 97		
3	Differential pulse polarography (DPP)	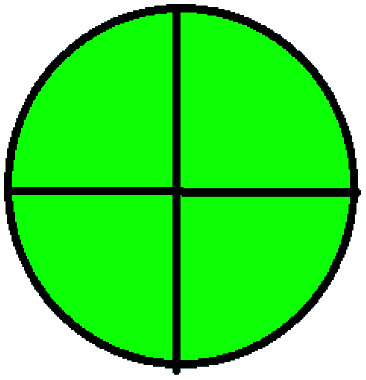	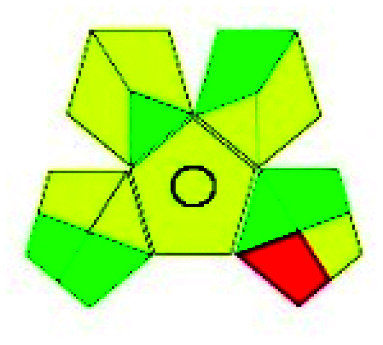	2 + 1 + 0 + 0 = 3	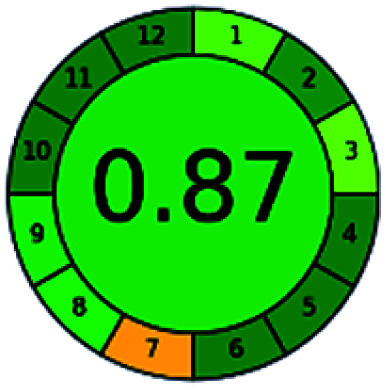	130
	0.01 M KH_2_PO_4_ buffer (pH 4.5)			ES = 97		
4	GCMS	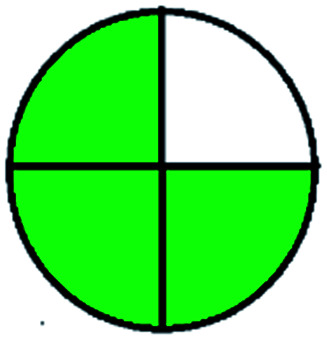	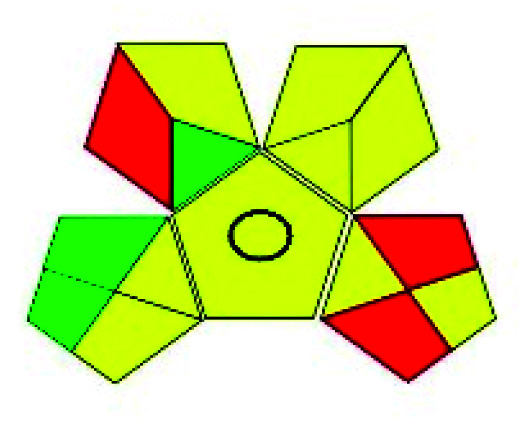	12 + 3 + 3 + 5 = 23	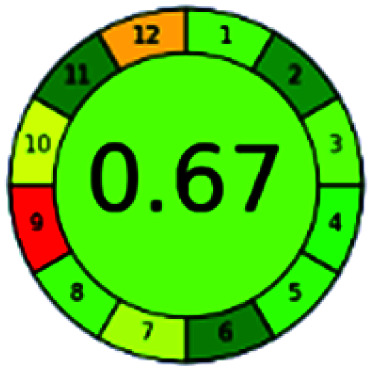	131
	Cold methanol, ethyl acetate, derivatization by using BSTFA			ES = 77		
5	PSMS	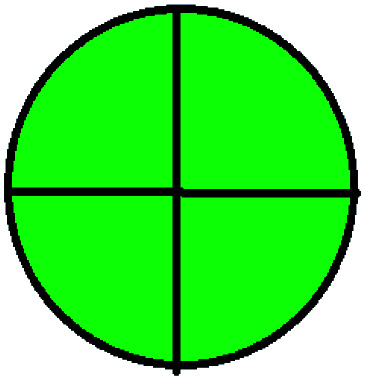	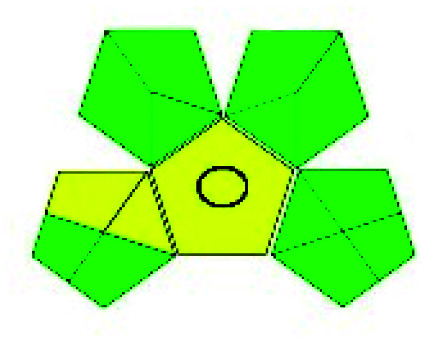	0 + 1 + 0 + 0 = 1	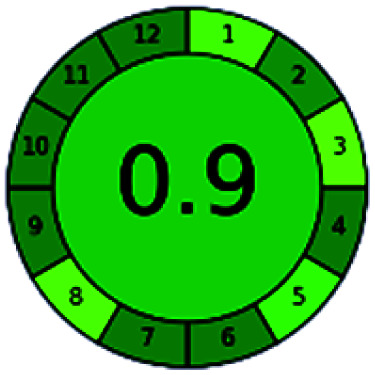	132
				ES = 99		
6	Microbiological technique	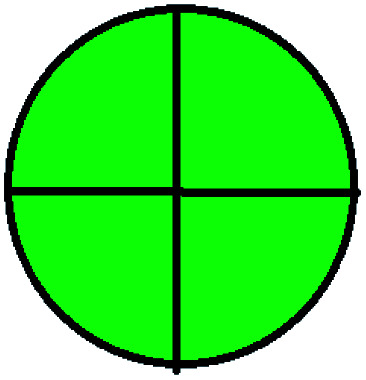	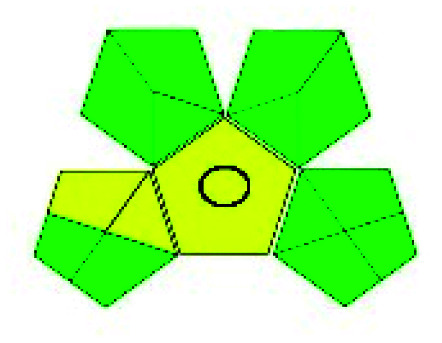	0 + 0 + 0 + 0 = 1	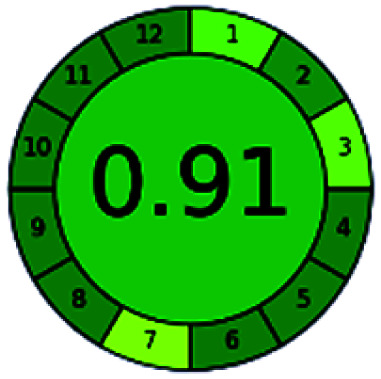	133
				ES = 100		
7	RP-HPLC	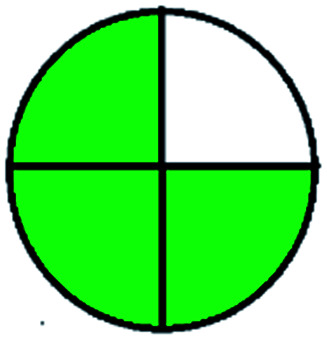	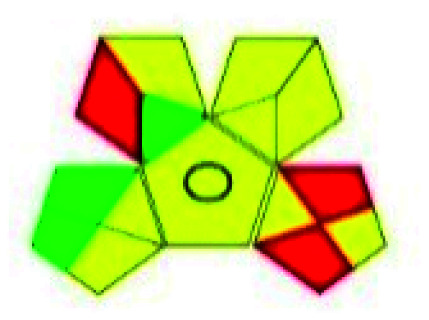	12 + 1 + 0 + 3 = 16	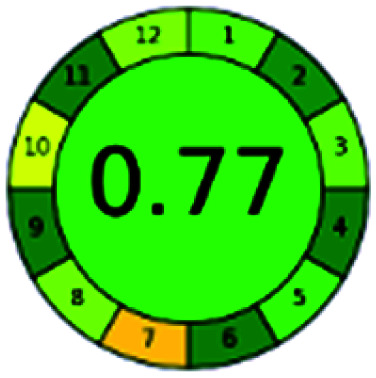	134
	AQbD			ES = 84		
	ACN and 0.05% acetic acid (pH 4) (50 : 50 v/v)					

## 4. Discussion

The present review compiles the analytical methods available for VOR estimation by spectroscopic, chromatographic, bioanalytical, and other techniques. The UV spectroscopic method mostly uses solvents like MeOH, water, HCl, and NaOH with a wavelength range of 252 to 256 nm, which shows that the method is effortless to use and accurate. The most eco-friendly approach was the one reported by Roy S. *et al.*^68^ This method utilized only water as a solvent for the analysis, making the technique more environmentally benign. Derivatization or photometric methods use different chemicals like tropaeoline ooo. Azo carmine-G has no serious toxic indication reported by any manufacturers in their data safety sheet, which shows that these chemicals shall be considered eco-friendly and can be used to determine other drugs. Assessment results also show that the method was eco-friendly related to the chemicals, but the number of steps involved in this method reduced the score towards its environmental friendliness.

**Fig. 5:**
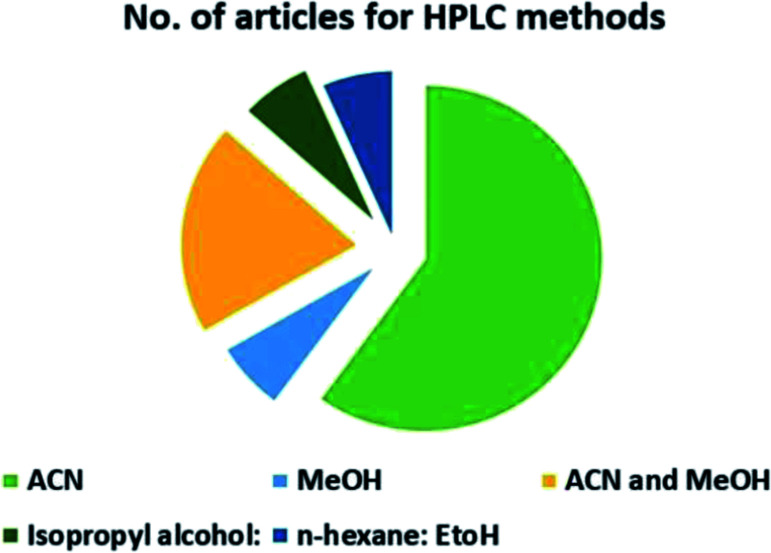
Organic modifiers used by reported methods in analyzing VOR in HPLC.

The HPLC methods utilized different solvents for the analysis of VOR in pharmaceutical substances. The eco-friendly method among the reported methods was Singh *et al.*^85^ In this, isopropyl alcohol and water was used as the mobile phase, which makes the process justifiable from an environmental standpoint. An approach can be picked based on the *R*_*t*_ as well. A method was developed by Lingamaneni K. *et al.*^72^ with an *R*_*t*_ of 3.02, which utilized less solvent compared to the other methods. The solvents used by the various reported methods in performing HPLC are depicted in Fig. 5. This indicates that most reported studies have utilized ACN and MeOH as solvents by changing the buffers at various pH levels. Among the fifteen reported methods four^73,78,80,83^ authors has employed the same mobile phase components, which showed the same green assessment results by changing the ACN : water composition. Adams *et al.*^74^ have used a mobile phase containing MeOH and triethylamine (TEA), in which TEA is considered toxic to human health and the environment as this reagent contains three pictograms indicating danger and has an NFPA score with three in both health and flammability, which leads to a decrease in the eco-score when applying the assessment tools. Nagarjuna *et al.*^75^ reported a method using ethanol and *n*-hexane as a mobile phase. Despite ethanol being considered an eco-friendly solvent, incorporating *n*-hexane made the method lose its environmental friendliness, as *n*-hexane contains four pictograms indicating danger. Both Huang *et al.* and Lingamaneni *et al.*^72,76^ have used a similar mobile phase composed of ACN and acetic acid. Still, variation in the assessment results was observed due to the run time and elution of the compound. As discussed earlier, the Lingamaneni *et al.* method had a better greenness profile than the other methods due to their run time. The remaining methods have utilized a common organic solvent such as ACN and MeOH along with a solid buffer. The different buffers used for the reported methods were phosphate with the corresponding salts of ammonium,^79^ sodium,^81,82,84^ and potassium,^86^ which produced similar results in the greenness assessment.

**Fig. 6:**
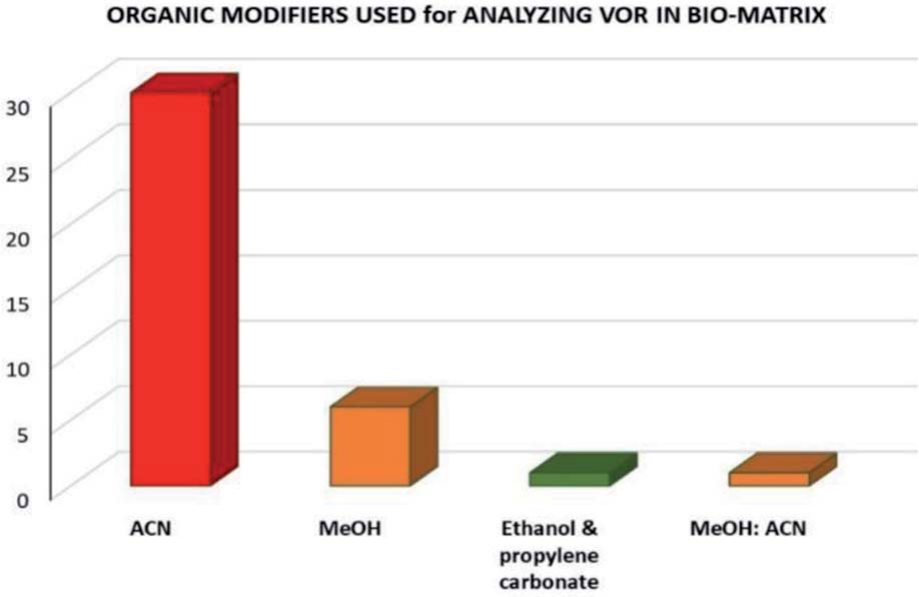
Organic modifiers used in the developed methods for analyzing VOR in biological matrixes.

Analysis of VOR and its combinations in biological fluids shows the importance of the drug and the need to develop an eco-friendly method without compromising the method quality as a better alternative to the reported approaches. The reported methods used several extraction techniques to separate VOR and its combinations such as biological matrix-like liquid–liquid extraction (LLE), protein precipitation, centrifugation, vortexing, simple mixing with the solvent, and direct injection. Among these extraction methods, direct injection and green extraction may help the technique be more environmentally sound and eco-friendlier. In the analysis of bioanalytical samples, the most preferred organic phase was again MeOH, ACN, and an appropriate buffer. The selection of the organic phase and the corresponding number of times it appears in the reported methods are shown in Fig. 6. According to this, ACN is considered as the predominant solvent used in most of the available methods, followed by MeOH. The present assessment tools showed that most methods had a similar greenness because the mobile phase selection was very similar with very slight variation.

Apart from the spectroscopy and chromatography methods, few reported methods explore the analysis of the drugs by applying the AQbD method for producing a long-term method, few other methods like sweeping-micellar electrokinetic chromatography, differential pulse polarography, paper spray mass spectrometry, and microbiological technique for the analysis of the VOR. The minimum linearity concentration reported was 2.49 ng mL^−1^ to the maximum of 100 μg mL^−1^.

The greenness was further analyzed using NEMI, GAPI, analytical eco-scale, and AGREE metrics for all reported methods, and the results are shown in Tables 1–5. As explained earlier, each tool used in assessing greenness follows a different method for performing the greenness assessment. Considering the reported methods, ACN is used to a greater extent than MeOH. Even though MeOH is less toxic when compared to ACN, only a few LC methods have been reported with MeOH and buffer as the mobile phase. Most methods mentioned using ACN as an organic modifier and other buffers show a repetitive technique that could be avoided. A green LC method has been reported to estimate VOR with the aid of eco-friendly solvents like propylene carbonate and ethanol and buffer with suitable method performance characteristics. There are very few green extraction processes utilized in the analysis of VOR in bio samples. Mainly the reported methods were composed of toxic solvents rather than green solvents.

The NEMI tool shows that two methods were eco-friendly among the spectrophotometric methods that used water and buffer as a solvent, one in HPLC methods that utilized isopropyl alcohol and water as mobile phase, no methods were green reported by HPTLC, and one in bio-analytical method that utilized propylene carbonate as a mobile phase. NEMI concludes that miscellaneous methods like sweeping-micellar electrokinetic chromatography, DPP, PSMS, and microbiological methods are safe and supportable for the environment.

The GAPI tool results strengthen NEMI's assumption in spectrophotometric, HPTLC, and bioanalytical methods, but picked a UFLC method as the best eco-friendly one among the reported ones; GAPI also indicates that the PSMS and microbiological methods are safest compared to the other methods concluded by the NEMI.

Pictographic assessment of AES is a numerical assessment tool that gives conclusive evidence with the help of a specific value. AES results support the inference given by GAPI for spectrophotometric, HPTLC, HPLC, and reported bio-analytical methods and strengthen it with a numerical value. In miscellaneous methods, AES showed a very slight edge toward the microbial method and showed a score of 100.

The final evaluation technique, AGREE, a very conclusive tool for green assessment, has confirmed the green-collar of the reported methods for greenness. AGREE has confirmed the method's greenness and supports AES's interpretation for spectrophotometric, HPTLC and reported bio-analytical methods but supported NEMI in the HPLC reported practices. Unlike AES, AGREE also showed a very slight edge toward the microbial process and showed a score of 0.95.

But prior consideration by four assessment tools states that the microbial examination method has the most eco-friendly results followed by the PSMS. The only drawback of these methods was that one consumes time. The other utilized only tears as a sample, nullifying the sampling procedure concept as it is not recognized as a transferable method. Only one bioanalytical method^125^ was eco-friendly, which utilized propylene carbonate as a solvent. The results indicate the importance of developing new green methods, which should be eco-friendly and easily applicable for industrial purposes.

Time is a crucial aspect for any industry in terms of production, as the quantity of production increases that directly enhances the effect of analysis time. So, it is always essential from an industrial point of view to consider an analytical method that can give the best results in less time by consuming less energy. Among the present methods for determination of VOR, the UV method consumes significantly less energy and time for analysis but has some flaws like reliability or reproducibility, whereas chromatographic methods are well adopted due to their several advantages. Among the chromatographic techniques, HPTLC is the most time and energy-consuming technique. LCMS and GCMS are the highly accurate methods for determinations but have a disadvantage like high energy consumption, which negatively affects the environment. The RP-HPLC is the most affordable and reliable in most industries for the analysis of pharmaceuticals, but this also has a disadvantage like energy consumption. Finally, the UPLC method is the most advantageous due to its highly reliable results in less time. Here, the application of time indicates an added advantage to the industries and reduces the generation of harmful greenhouse substances into the environment.

## 5. Conclusion

According to the current review, the methodological approaches accessible for VOR determination comprise spectrophotometry, chromatography, and biological test methods. Each method has its own set of advantages and disadvantages when compared to others. As methods have been continuously developed to assess VOR, this drug's importance shows a need for a non-polluting methodology for drug analysis. Among the methods reported, only one approach has utilized more environmentally supportable practices, an introductory note for future research regarding this compound. Analysts and expert formulators must create more environmentally friendly techniques for estimating VOR that use less hazardous solvents. Additional LC-MS/MS-based methods may quantify the medication in biological matrixes, which might be more critical for VOR therapeutic monitoring. Analytical quality by design, which was performed using Box–Behnken design, shows some supremacy among the methods due to long-term sustainability. AQbD deals with developing strategies for the future, but the drawback of the methods used to determine VOR is that they have not applied bio-degradable solvents. Therefore, incorporation of quality (AQbD) and eco-friendly (GAC) principles in developing techniques is highly recommended for the estimation of VOR in various matrixes, and more methods need to be developed to analyze VOR, which should be based on clean green analytical chemistry and make the environment and environment analyst safe.

## 6. Future aspects

The pharmaceutical and chemical industries are concerned about environmental safety and green analytical method development as a value in the vision of pharma industries 4.0. There is an opportunity to develop environmentally benign methods more positively by applying greener analytical techniques in analytical research and development *via* regular quality control activities. The future development of a stable green HPLC method with the aid of quality by design to estimate VOR without the need for revalidation would be more beneficial. There were no green HPLC methods for estimating VOR and its known and unknown impurities, and the same should be developed. The new transformation of extractions included using lower organic solvents, sorbents, better extraction and clean-up, fewer pre-treatment steps for a sample, and improved selectivity adoptions into the method development of bioanalytical matrixes more eco-friendly.

The new transformation of extractions included the usage of lower organic solvents, sorbents, better extraction and clean-up, fewer pre-treatment steps for a sample, and improved selectivity. Green microextraction technology is a user-friendly platform for analysts and far less environmentally damaging and provides even fewer toxic solvents, miniaturization, greater automation, and online coupling power with analysis techniques. Adopting these technologies for pharmaceutical analysis makes the method more stable, environmentally benign, and lasts longer.

Furthermore, no quantitative IR or NIR method was reported to estimate VOR, which should be developed and shall be the greenest method over LC and other methods and delivers the scope of developing quantitative IR methods to assess VOR.

## Conflicts of interest

There are no conflicts to declare.

## Supplementary Material
